# Anti-bacterial and anti-inflammatory properties of *Vernonia arborea* accelerate the healing of infected wounds in adult Zebrafish

**DOI:** 10.1186/s12906-024-04383-8

**Published:** 2024-02-19

**Authors:** Lalitha Vaidyanathan, T. Sivaswamy Lokeswari

**Affiliations:** 1 Department of Biomedical Sciences, Sri Ramachandra Institute of Higher Education and Research, Chennai, Tamil Nadu India; 2 Department of Biotechnology, Sri Ramachandra Institute of Higher Education and Research, Chennai, Tamil Nadu India

**Keywords:** Wound infection, Zebrafish, Neutrophil, Myeloperoxidase, Interleukins, Inducible nitric oxide synthase

## Abstract

**Background:**

Management of wounds and healing under impaired conditions are the major challenges faced globally by healthcare workers. Phytocompounds which are anti-microbial and capable of modulating inflammation contribute to overall wound healing and regain of the lost structure and function especially in wounds impaired with polymicrobial infection.

**Methods:**

An acute cutaneous impaired wound model using adult zebrafish was validated to simulate mammalian wound pathophysiology. This model was used to evaluate phytofractions of *Vernonia arborea* in the present study, for reduction of infection; myeloperoxidase (MPO) as a marker of infection; neutrophil infiltration and resolution; kinetics of inflammatory cytokines; and wound repair kinetics (viz., nitrite levels and iNoS expression; reepithelisation).

**Results:**

Four fractions which were active in-vitro against five selected wound microbes were shown to reduce ex-vivo microbial bioburden upto 96% in the infected wound tissue. The reduction in CFU correlated with the neutrophil kinetics and MPO enzyme levels in the treated, wound infected zebrafish. Expression of pro-inflammatory cytokines (IL-6 and TNF-α) was downregulated while upregulating anti-inflammatory cytokine (IL-10), and nitric oxide signalling with fourfold increase in iNOS expression. The adult zebrafish wound model could well serve as a standard tool for assessing phytoextracts such as *V. arborea* for wound healing with anti-microbial properties.

**Graphical Abstract:**

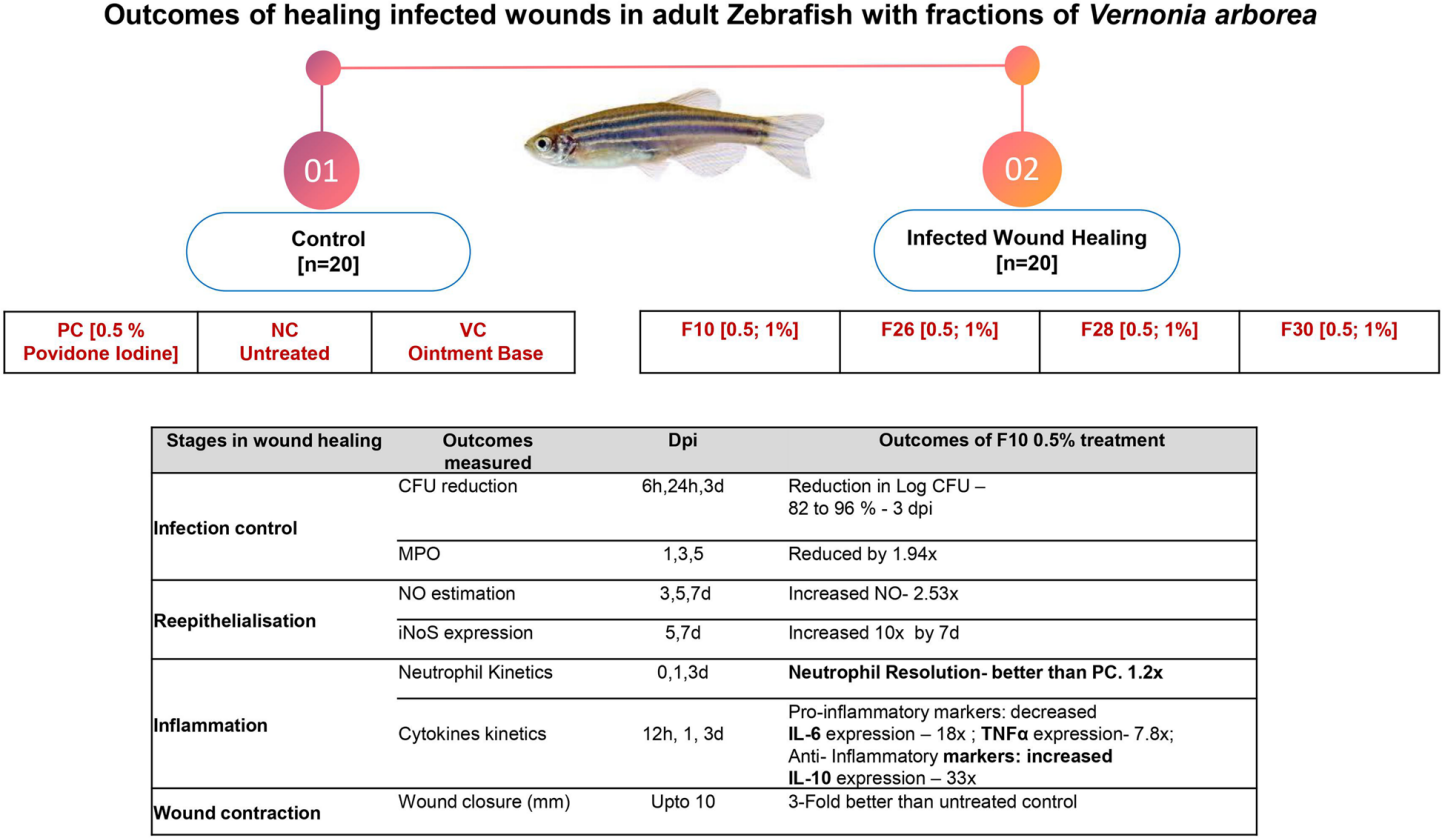

**Supplementary Information:**

The online version contains supplementary material available at 10.1186/s12906-024-04383-8.

## Background

Infection by polymicrobial community is one of the major characteristic features of chronic wounds [1]. Though mere microbial colonization does not impair healing, there is a delay in healing of chronic wounds with transition to infection in comorbid conditions. Bacteria in polymicrobial environment pose more resistance than when they occur alone,with the microbial synergy contributing to quorum sensing and effectively altering the gene expression in the host tissue [2]. We studied polymicrobial (5 prevalent bacteria) infected wound healing which has not received much attention in the literature. Such infections were reported to be extremely multidrug resistant naturally and recorded with up to 69% mortality rates [3, 4] caused by redundant use of antibiotics. Wound management thus involves the rationale use of drugs/phytocompounds/ regenerative therapies, in addition to wound debridement and infection control plans. In studies screening for such anti-microbial agents, the determination of the MICs of useful compounds and their ability to reduce the colony forming units (CFU/mL) at the wound site in various wound models were essentially reported [5, 6]. Identifying natural compounds that could have microbicidal and inflammation-modulating properties could accelerate wound management by recreating the favourable microenvironment and facilitating the repertoire of cellular events [7]. About 450 plant species that modulate one or more of the stages of the wound healing process were identified to aid wound management [8, 9] including species of *Vernonia*. The phytochemicals were diverse in structure with diverse properties including flavonoids, anthocyanins, quinones, cinnamic acids, terpenoids, phytosterols, phenolic compounds, alkaloids, saponins, carbohydrates, glycosides, tannins among others [10]. The Supplementary Tables S1 & S2 report over 10 *Vernonia* species with biological activities (for extracts or known compounds) such as anti-microbial, anti-inflammatory, anti-oxidant, wound healing, anti-diabetic, anti-cancer among others. One of the little investigated species in wound healing includes, *V. arborea* Buch.-Ham., the only tree species of the family Asteraceae, found in the Western Ghats of India and Sri Lanka. A decoction from this tree species, *V. arborea* traditionally promoted wound healing, of mouth ulcers, and was given to women after child birth [11, 12].

Several species of the genus *Vernonia* showed inhibition of Gram positive and Gram negative bacteria contributing to wound management (Supplementary Table S3 shows their MIC and MBC concentrations). The hexane leaf extract of the plant, *V. arborea* contained phytoconstituents like the sesquiterpene lactones, active against plant pathogens [13] and the phytoconstituents present in the hexane leaf extract of *V. arborea* contained phenols, steroids, terpenoids and tannins as given in supplementary Table S4. These extracts showed in vitro anti-bacterial activity against five selected wound pathogens included in the present study (Supplementary Table S1) [14].

The methanol and aqueous extracts obtained from the leaves and bark of *V. arborea* were tested for wound healing activity in the exision, incision and dead space wound models of Swiss Wistar rats using the ointment formulation [15, 16]. In comparison with the untreated controls, both the extracts promoted faster epithelialisation of the wound, 17.86 days and was comparable to the positive control (16.15 days,1% w/w framycetin sulphate cream ointment). The extracts showed two-fold increase in the skin breaking strength and high hydroxyproline content compared to the control group. Thus, wound healing potential of *V. arborea* extracts in rat models supported the choice of the plant for our studies.

Wound models used to screen healing potency of novel substances in in vivo animal models include human, rodents, rabbit and porcine models. However, a difference in the healing of full-thickness wounds in adult mammals with scarring as opposed to regeneration of new hair follicles in lab mice [17] points to the need for validating novel models such as adult zebrafish. Naomi [18] showed that, “The basic principles of the wound-healing mechanism are conserved between humans and zebrafish due to the similarity of their skin structure.” They are considered as viable in vivo models for cutaneous wound healing, angiogenesis and regeneration studies in drug discovery. About 4–5 studies used adult zebrafish in healing of wound caused by amputation or laser with interventions (*Spirulina maxima* based pectins [19], *Curcuma longa* extracts [20], Silver nanoparticles [21]) and none were with acute cutaneous excision wound model. While zebrafish has its drawbacks in the mechanism of drug/plant extract metabolism as compared to mammalian systems, the ease of experimentation and similarity of the cutaneous wound healing process can still be exploited to discover novel agents such as *V. arborea* extracts. The anti-inflammatory (modulation of neutrophil infiltration at the wound site) and anti-oxidant (maintaining redox balance in cells) potency of the *V*. *arborea* fractions were demonstrated in the adult zebrafish, acute cutaneous excision wound model [22]. The plant fractions with such in-vitro anti-microbial potency and in-vivo anti-oxidant, anti-inflammatory activities also showed 93% cell migration in HaCaT (human keratinocyte) cells in-vitro (unpublished data).

Impaired wounds caused by bacterial infections in Zebrafish are invaluable for rapid, direct observations especially for evaluating novel healing agents for their cellular, biochemical and molecular activities in wound healing [18, 23]. Monitoring each phase of healing using cellular and molecular markers in the *V. arborea* treated in-vivo adult zebrafish infected wound models was the major objective of the present study. The bioactivity of the fractions was evaluated for the first time, for reduction of infection, myeloperoxidase (MPO) as a marker of infection; neutrophil infiltration or resolution; kinetics of inflammatory cytokines; and wound repair kinetics (viz., nitrite levels and iNoS expression). This in-vivo study model under impaired wound conditions could facilitate screening novel wound healing agents, or even understand the mechanisms of action of such agents.

## Methods

### Selection of wound microbes

The test strains were selected based on their prevalence in the clinical wounds [24–28]. The selected test strains were obtained from ATCC and maintained in the lab on LB agar or Hichrome selective agar media. Strains used include *Escherichia coli* (ATCC 25922), *Staphylococcus aureus* (ATCC 25923), *Pseudomonas aeruginosa* (ATCC 27853), *Klebsiella pneumoniae* (ATCC 27736) *and Stenotrophomonas maltophilia* (ATCC 13637).

The bacteria were maintained in fresh LB broth at 37 °C for 18 h. Cells were washed with PBS (phosphate buffered saline, pH 7.4) and CFU was adjusted to 5 × 10^5^ and used in the assays.

### Bioactive fractions of V. arborea

The *Vernonia arborea* leaves were collected from Anaimalai Hills, Pollachi District (10°22′N 77°07.5′E). The collected material was identified and authenticated by Prof. P. Jayaraman, Director, Institute of Herbal Botany, Plant Anatomy Research Centre, Chennai, India and a voucher specimen maintained in the department (PARC/2012/1392). Dried leaves (1 kg) of this tree species collected in October were extracted in hexane. Four fractions (F10, F26, F28 and F30) obtained from column chromatography of the hexane leaf extract of *V. arborea* were identified with anti-microbial activity against the selected test microbes and their MICs determined using Resazurin Microtiter Assay (REMA) [14]. The four bioactive fractions, F10, F26, F28 and F30 were prepared as an ointment for topical application on the infected, cutaneous wounds in adult zebrafish. The ointment was prepared at 0.5 and 1% w/w concentrations by mixing with warm petroleum jelly.

### In-vitro time kill kinetics of bacteria for the bioactive fractions

To determine the time for reduction of populations in-vitro, the time kill assay for the four fractions at half MIC, MIC and twice MIC was performed with all the test strains. Suspensions of the test strains with inoculum strength of 5 × 10^5^ were treated with the fractions. Samples from the treatment tubes were plated at 0, 2, 4, 6, 8, 10, 20 and 24 h. Tubes with untreated bacterial suspension and suspension treated with ampicillin at inhibitory concentrations served as the negative and positive controls. The colonies were counted after 24 h incubation and the CFU/mL were determined. Triplicates were maintained and a graph was plotted with the log CFU/mL values against observation time [29]. The log reduction in CFU was calculated as:

$${\mathrm{Log}}_{10}\;\mathrm{reduction}\;\mathrm{of}\;\mathrm{CFU}=\left[{\mathrm{Log}}_{10}\left({\mathrm{CFU}}_{\mathrm{sample}}/{\mathrm{CFU}}_{\mathrm{control}}\right)\right]$$

### Establishment of cutaneous wound infection, its treatment and tissue sampling

Healthy wild type adult Zebrafish of about 5–7 months were randomly grouped into control and experimental groups. The average weight of the fish was 0.4–0.6 g with 50:50 male:female ratio maintained. Quick mechanical cutaneous wound (OD 3 mm; depth 2 mm) was established in the dorsal side of the anaesthetised adult Zebrafish using a semi-automated device [22]. The established wound was infected with 20 µL of the polymicrobial suspension (*E. coli*,* S. aureus*,* P. aeruginosa*,* K. pneumoniae and S. maltophilia*) with a total CFU of 5 × 10^5^ cells/mL. The suspension was mixed with the ointment base (or along with the test fractions) and applied to the wound area [30].

The wounded fish were grouped into three control and four experimental groups with 20 numbers in each. Positive control group received 0.5% povidone iodine ointment, untreated control group received no treatment, vehicle control group received the ointment base. The four experimental groups received 0.5% and 1% w/w ointment formulation of the four bioactive fractions, F10, F26, F28 and F30. The fish were taken to the experimental tank after recovery and were observed for any topical lesions or allergies other than the wound area. These wounded, infected and treated fish were used to determine reduction in CFU/mL and the inflammatory kinetics using histopathology, tissue markers, biochemical markers and immunomolecular markers.

#### Anesthesia and euthanasia

Zebrafish brood stocks were collected from approved vendors, from Kolathur farms, Chennai, TamilNadu, India and maintained in the lab. The fish were anesthetised using Tricaine Methanesulfonate (MS-222; Sigma) during the experimental procedures as recommended by Zfin.org. Stocks of Tricaine solution (0.4% in 1 M Tris, pH 9) stored at 4 °C was used to prepare working solution by diluting 4.2 mL in a 100 mL clean tank water just before use. The fish lost movements in Tricaine in about 1.5 min, sustained for about 2–3 min and were moved to a recovery tank (fresh tank water) and monitored for revival of movements, which took about 3–5 min. The fish were wounded between these time periods. The fish in the recovery tank were observed for restoration of active movements and returned to the experimental tanks.

The fish were euthanised by exposing to overdose of Tricaine Methanesulfonate (200–300 mg/L); held immersed for about ten minutes leading to subsequent death by hypoxia [31]. The procedures were approved by the Institutional Animal Ethics Committee (XXXXII/24th January 2015).

#### Tissue sampling

The tissue samples around the wounds were collected immediately after euthanizing and frozen at -20 °C for RNA extraction and further analysis. The samples for wound infection studies were immediately taken in a tube containing 1 mL sterile saline. The microbes were brought to suspension using a cyclomixer before further analysis.

### Determination of neutrophil infiltration

Microtome sections of the infected wound tissue were prepared. The sections were stained with Hematoxylin and Eosin (H&E) stain to count the neutrophil population at 0 h post infection (hpi), 1 and 3 days post infection (dpi) [20, 32].

### Measurement of reepithelialisation and granulation tissue formation

The degree of migration of the cells to the wound site were observed in the fish by tracing the outer diameter of the wound margin and recorded at various time intervals of treatment, 0, 5, 7 and 10 dpw. Reduction in wound diameter indicating migration  of keratinocytes and epithelial cells near the wound margin to cover the wound was observed. This observation gave the rate of reepithelialisation and wound closure. Granulation tissue formation was observed in terms of appearance of re-stratified epithelial layer along the wound surface [33].

### Quantification of MPO in the infected wound tissue

MPO levels (as a marker of infection) in the infected wound tissue were measured spectrophotometrically. The infected wound tissue was excised and known weights homogenised with Phosphate Buffered Saline. The supernatant was mixed with substrate solution containing Guaiacol and H_2_O_2_. Tetraguaiacol was estimated at 470 nm [34, 35] indicating the MPO levels in (mUnits/mL) on 1, 3 and 5 dpi.

### Determination of nitrite levels in infected wound tissue

Known weights of wounded tissues were homogenised in lysis buffer and the diluted supernatants were mixed with Griess reagent (Sigma). Absorbance at 548 nm were estimates in µmole/mg of standard nitrite [36] on 3, 5 and 7 dpi.

### Ex-vivo anti-bacterial activity

The CFU reduction in the wound infected tissue was studied in the control and treated groups at 6 h, 1 and 3 days post infection (dpi) (shown as bar charts). The percentage CFU reduction was tabulated across the control groups and the treatment groups (3 dpi) using the following formula:

$$\small \mathrm{Percentage \,reduction}\hspace{0.17em}=100\ \mathrm{ x }\left(\mathrm{CFU \,of \,control}-\mathrm{CFU \,of \,treated \,group}\right)$$

Individual bacteria from the polymicrobial sample were identified using HiChrome selective agar media.

### Gene expression analysis

Quantitative Reverse Transcriptase Polymerase Chain Reaction was performed with the infected wound tissue at specific observation times to study the measure of relative fold change in expression of the genes associated with wound inflammation and repair. Total RNA was extracted from the infected wound tissue using RNeasy Mini Kit (Qiagen) and the RNA quality was confirmed by determining absorbance ratio at 260/280 nm and quantified using nanodrop (Thermofisher). Gene expression pattern was analysed using the One Step Primescript III RT-qPCR Kit (TAKARA) normalised to β-actin, the housekeeping gene [37]. Relative fold change was computed using the ∆∆CT method [38].

Using Zebrafish gene specific primers the expression levels of pro-inflammatory cytokines, IL-6 [39], TNF-α [37] and the anti-inflammatory cytokine IL-10 [37] were measured in the wound infected tissue at 12 h, 1 and 3 dpi. The fold change in iNOS [40] expression was measured in the control and treated groups on 5 and 7 dpi [41].

### Statistical analysis

Data were analysed with IBM.SPSS statistics software 23.0 version. All data are presented as mean ± standard of the mean (SEM). Multivariate analysis was performed using the Kruskal Walli’s test and for bivariate analysis, the Mann–Whitney U test was used. *P* < 0.05 and 0.01 was considered statistically significant. One-way analysis of variance (ANOVA) followed by Dunnett’s post hoc test was used to compare the population decrease in the time kill assay.

## Results and discussion

Phytochemicals with microbicidal and inflammation-modulating properties were most often reported in wound healing studies [42–44]. Wound healing with *V. arborea* was reported using extracts of the bark or leaves in excision, incision, and dead space wound models of Wistar rats [15, 16] wherein the healing outcomes at the end of 6–10 days post wounding were compared. In this study we evaluated the healing ability of *V. arborea* extracts in adult zebrafish cutaneous wound model from day 0 as it mimics the mammalian wound pathology [18] under normal and impaired conditions.

*V. arborea* extracts (F10, F26, F28 and F30 fractions) were anti-microbial for the five wound microbes tested here and their MICs were determined in REMA microbroth dilution assays [14]. The potency of the bioactive fraction to enhance wound repair kinetics in an infection model were quantified based on:

i) reduction in infection [viz., in-vitro time-kill kinetics; ex-vivo CFU reduction; neutrophil resolution; MPO as a marker of infection]
ii) kinetics of immuno-molecular markers [viz., expression of pro-inflammatory markers: IL6; TNF α and anti-inflammatory markers: IL-10]
iii) wound repair kinetics (viz., nitrite levels and iNoS expression) & reepithelization.

### Reduction in infection

#### In-vitro time kill kinetics

**Fig. 1:**
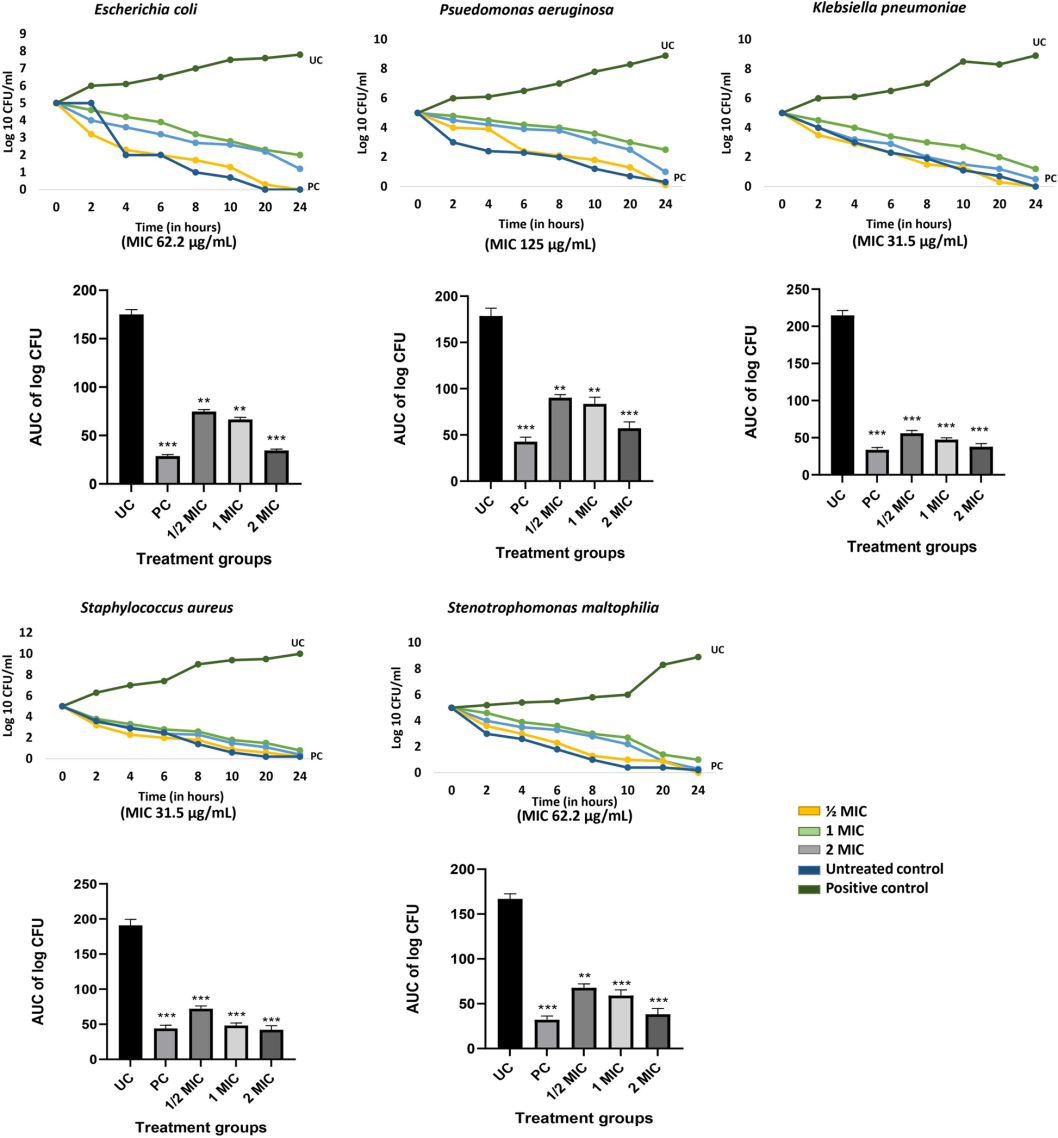
Time kill kinetics of *V. arborea* Fraction 10 against all five test strains with the respective MIC and AUC of log CFU shown below. UC, untreated control; PC, positive control (ampicillin at inhibitory concentration); *n* = 3; values are mean ± SEM. ** *p* < 0.05, *** *p* < 0.01 compared to the untreated control (one-way ANOVA followed by Dunnett’s post hoc test)

Time kill curves of the five test strains with the four fractions F10, F26, F28 and F30 are shown in Fig. 1 for F10 & Supplementary Figures for other fractions (Fig. S1, S2, & S3). The test fractions showed reduction in CFU in comparison to the untreated control. Fraction 10 showed 90% reduction in CFU at 1 MIC for the five strains tested. The MICs for the four fractions against each of the five microbes determined by REMA assay [14] are given in parenthesis on the X-axis.

*E. coli* showed upto 4.1 fold reduction in log 10 CFU when treated with F10 at 62.2 μg/mL. *S. aureus* was very sensitive to F10, F26, F28 and F30 fractions and showed 12.5 fold reduction in log populations within 24 h at 31.5 μg/mL. The reduction was equivalent to that observed after treatment with the positive control. *S. maltophilia*, was sensitive to F30, with the maximum reduction of about 16 fold from 0 to 24 h at the least concentration of 15.62 μg/mL while with F10 similar reduction at a concentration, 62.5 μg/mL was observed. At 125 μg/mL, F10 and F30 exhibited nearly 3–fourfold reduction in log 10 CFU of *P. aeruginosa*, the most resistant test microbe. The log 10 CFU of *K. pneumoniae* reduced tenfold upon treatment with F10 and F30 compared to the other fractions. Having shown this reduction in population with the four fractions, the populations in the treated and infected wound sites was then evaluated ex-vivo.

#### Ex-vivo reduction in polymicrobial load at the wound site

The efficacy of *V. arborea* F10 extract in healing of acute wounds (uninfected) in adult Zebrafish was earlier reported [22]. The fractions were also efficient at reducing microbial populations by 90% of the five test strains in time kill assays (Fig. 1 & Supplementary Figures S1, S2, S3 & S4). Extending this study using infected wounds in the in-vivo zebrafish is a novel approach as one of the causal factors of transforming acute wounds to chronic non-healing wounds is wound infection.

**Fig. 2:**
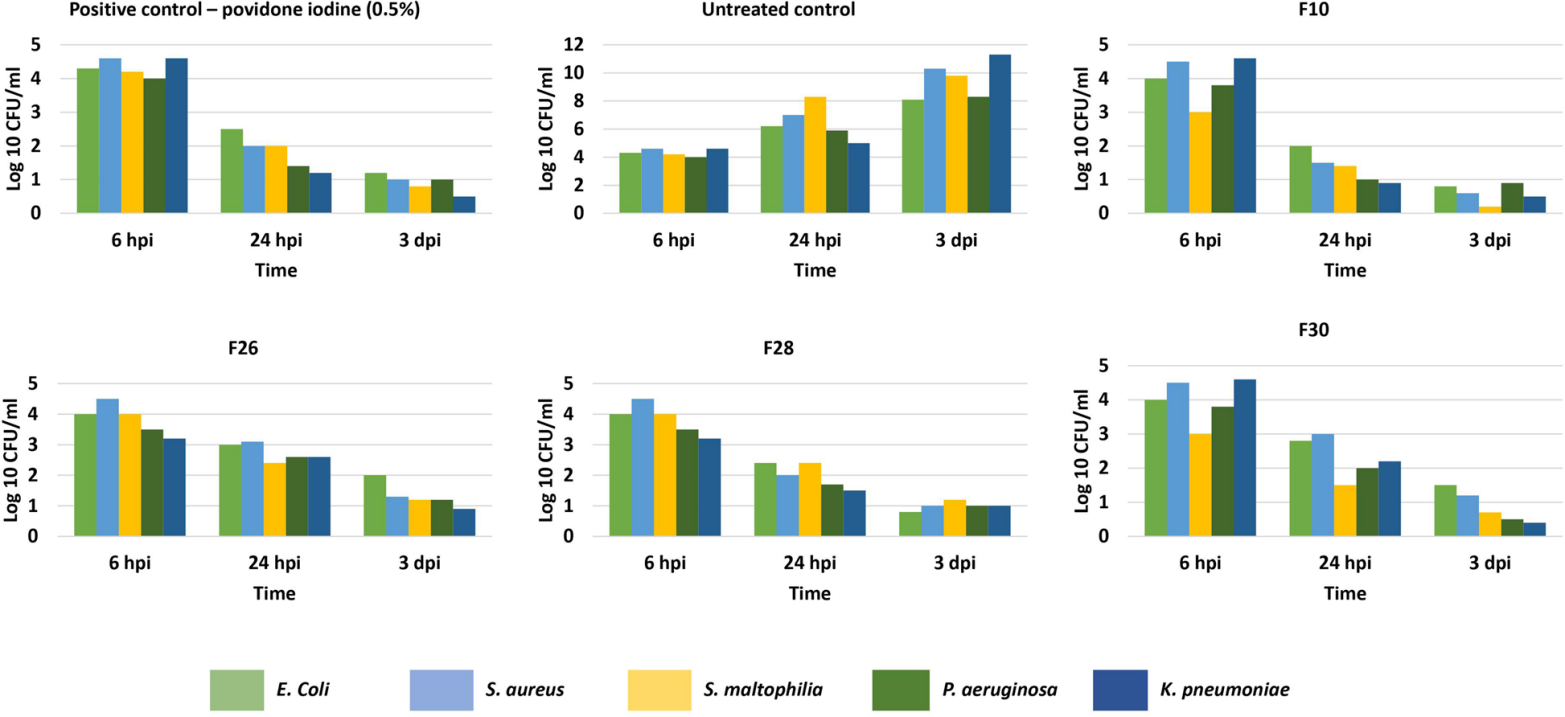
Bacterial populations in the zebrafish infected wound tissue at 6, 24 hpi and 3 dpi. Treatment with bioactive fractions (0.5%) showed a reduction in CFU comparable to the positive control. The untreated control showed a constant increase in CFU with time. Values are mean [*n* = 20] ± SD, *p* < 0.05, *p* < 0.01 compared to positive or untreated control, respectively

The microbial load in the wound tissue was measured as % CFU reduction on 3 dpi (Table 1), marking the efficiency of the clearance by inflammatory neutrophils. Test fraction treatment at 0.5% w/w ointment, reduced CFU in the infected wound tissue ex-vivo, several folds compared to the untreated tissue (Fig. 2). Maximum of 96% reduction in CFU was observed in the F10 treated group (Table 1).

**Table 1:**
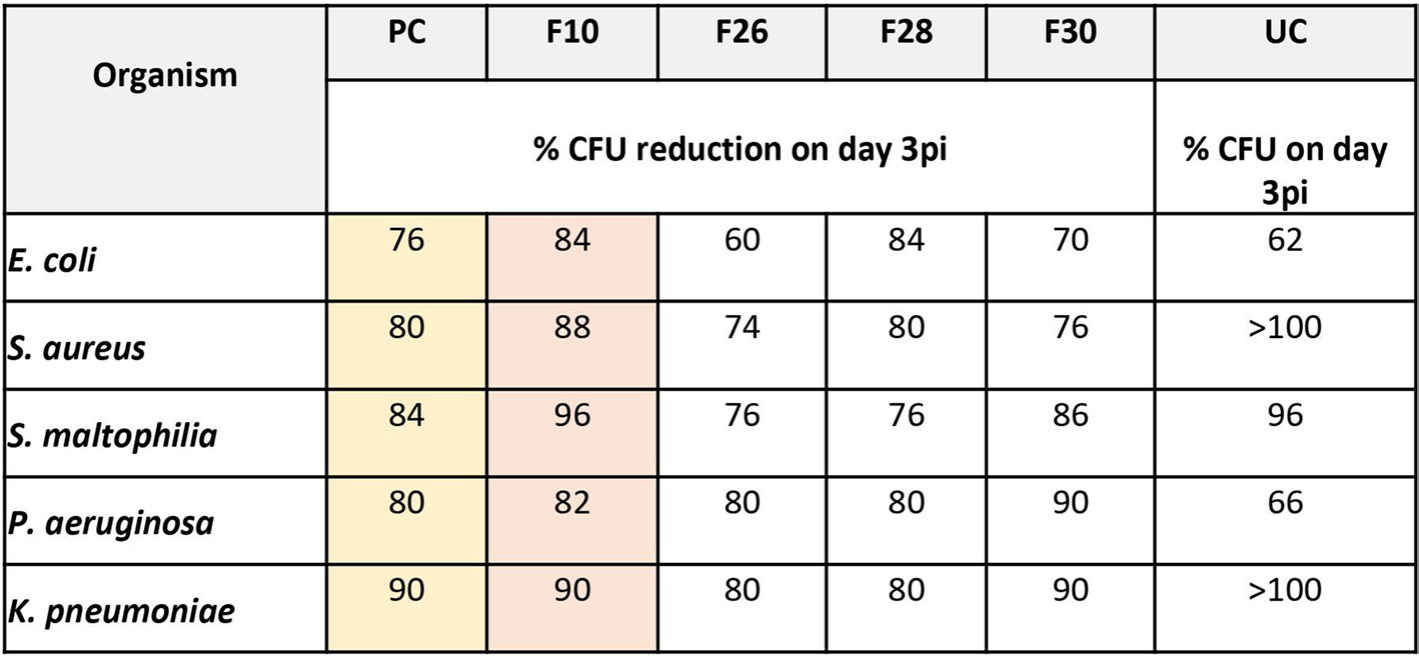
Percentage CFU reduction observed in zebrafish experimental and control groups treated with 0.5% of *V. arborea* test fractions 3 dpi. PC, positive control; UC, negative control

The results correlated with a study on anti-bacterial activity of Mupirocin in reducing bacterial CFU in the *S. aureus* infected superficial abrasion mouse model. The maximum infection load was ≥ 10^5^ CFU/mL and there was a 6 log_10_ reduction in CFU upon treatment indicative of bactericidal activity [45].

The current study is the first report of an infection-induced impairment model of acute cutaneous excision wound in the adult zebrafish. The subsequent reduction in the bacterial bioburden upon treatment with bioactive fractions from *V. arborea* are reported for the first time. These results correlated with the neutrophil infiltration and clearance profile and reduction in MPO levels, the enzyme marker for infection, in the infected wound tissue.

#### Neutrophil resolution in infected wound tissues

Among the cells belonging to the innate immunity, the neutrophil population is the major group that take part in tissue inflammation [46] induced during wounding. The inflammatory phase is essential to clear the microbial load at the wound site.

**Fig. 3:**
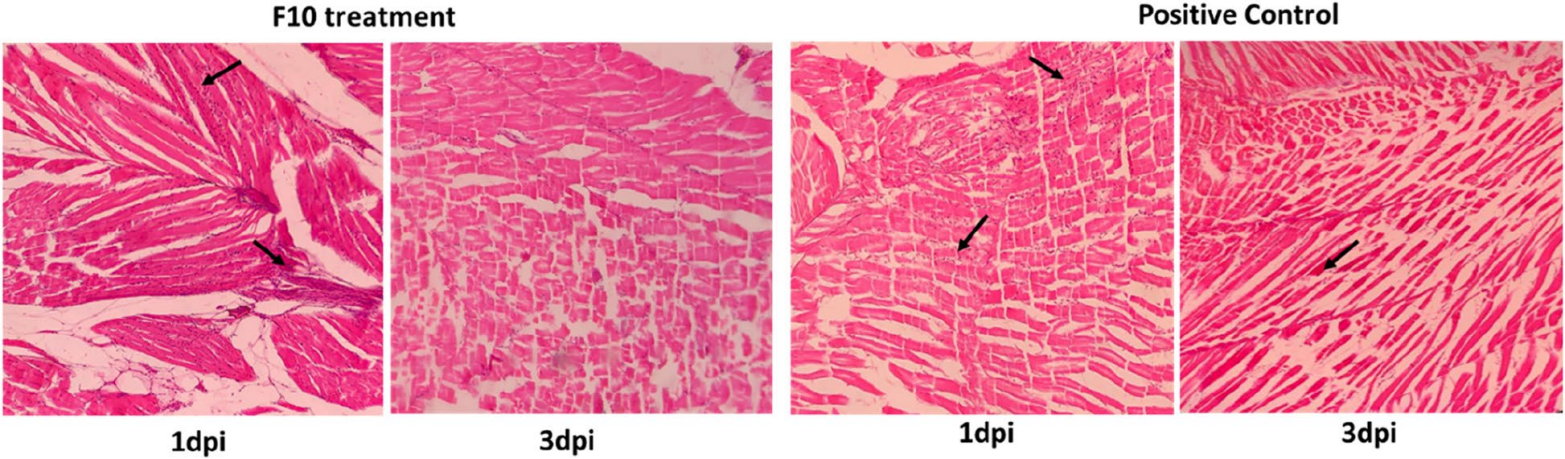
H&E stained sections of the zebrafish infected wound tissue showing better resolution of neutrophil population [*n* = 20] 3 dpi after treatment with *V. arborea* F10 (0.5%) in comparison with the positive control

**Fig. 4:**
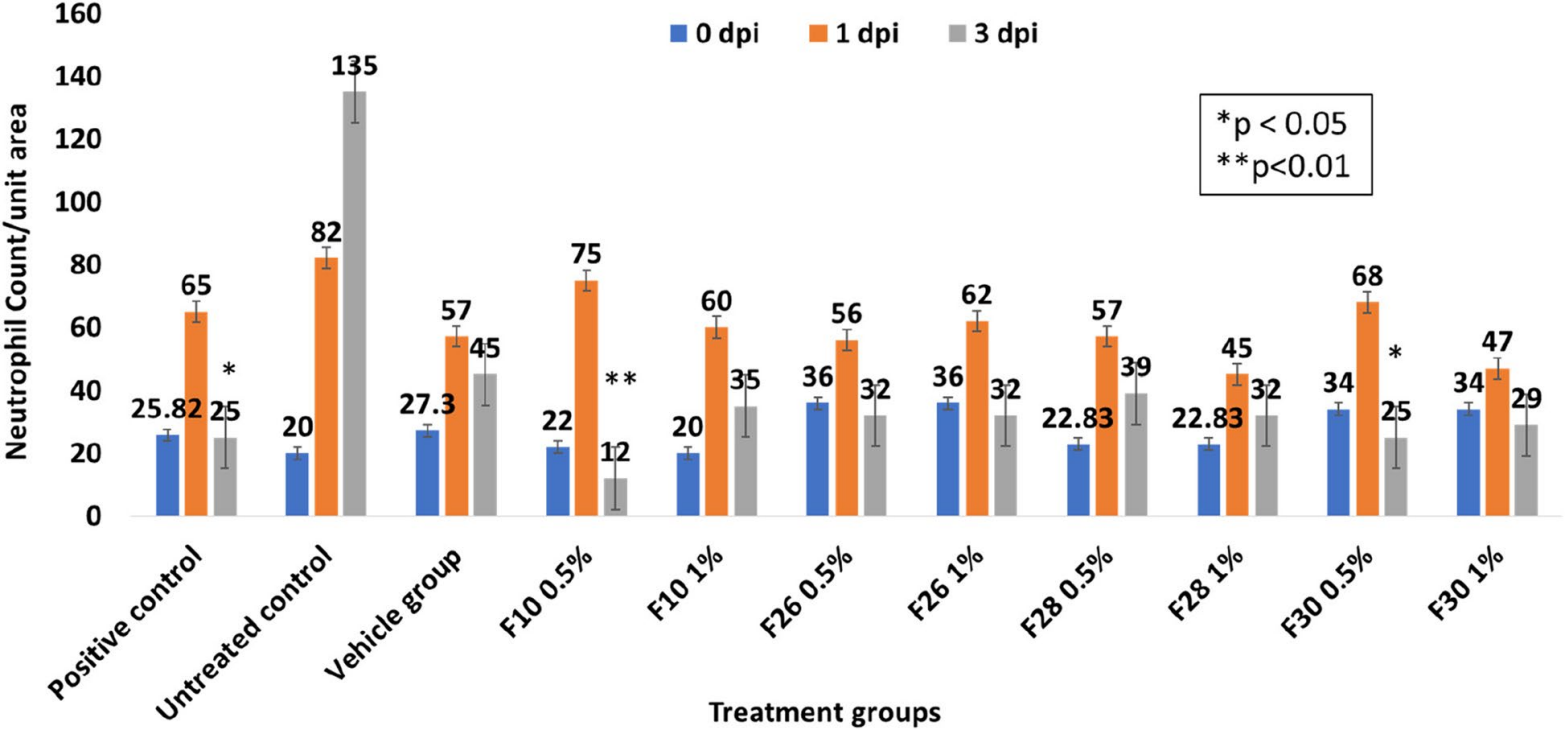
Neutrophil population at the zebrafish infected wound site recorded on 0, 1 and 3 dpi. The bioactive fraction treated wounds exhibited high infiltration on 1 dpi and good resolution on 3 dpi. *V. arborea* F10 at 0.5% treatment showed maximum resolution of neutrophil population in comparison to the control. Values are mean [n = 20] ± SD, *p < 0.05, **p < 0.01 compared to positive or untreated control, respectively

Two concentrations (0.5% and 1%) of each extract, F10, F26, F28 and F30 were applied. The results are presented in Figs. 3 & 4. The infected wound, treated with the F10 (0.5%) showed optimal neutrophil infiltration and threefold better resolution compared to untreated group. There was a 6.25 fold reduction in neutrophil population from day 1 to 3 in F10 treated group while the positive control showed 2.6 fold resolution (Figs. 3 and 4).

Resolution of inflammation was much earlier post treatment in infected wound tissues as evidenced by the neutrophil profile. The neutrophil clearance 3 dpi correlates well with the reduction in bacterial burden in treated groups (Fig. 2, Table 1). Such a resolution of inflammation when the hypoxia-inducible factor-1α (Hif-1α) reduces neutrophil apoptosis and reverses migration was shown in Zebrafish inflammation model [47].

#### MPO as a marker of infection

**Fig. 5:**
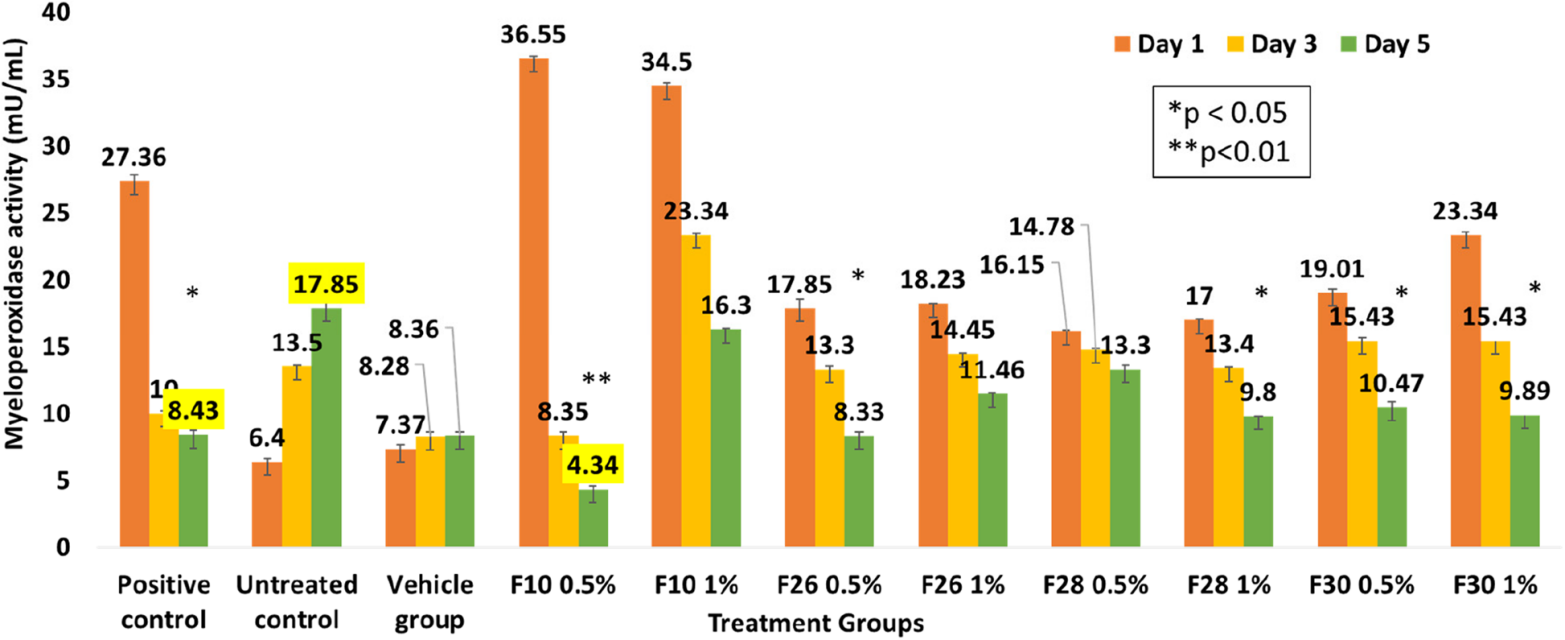
Myeloperoxidase enzyme activity measured across the zebrafish control and treatment groups on 1, 3 and 5 dpi. Values are mean [*n* = 20] ± SD, *p < 0.05, **p < 0.01 compared to positive or untreated control, respectively

MPO, a marker of infection is secreted by the neutrophils in response to the microbial load and their levels indicate the persistence of infection in the tissue. Infected wounds treated with 0.5% and 1% ointments were excised for the assay. There was a significant reduction in the MPO level from 3 to 5 dpi in the positive control and the test groups (Fig. 5). F10 reduced the level by 1.94 and 4.11 folds compared to positive and untreated controls 5 dpi. MPO levels in the infected wound tissue and treated tissues correlated with the neutrophil population (Fig. 4) and reduction of infection (Table 1) in wound tissue.

Geraniin, a major constituent of *Phyllanthus muellerianus*, reduced MPO expression in wound tissue in rat excision wounds [48]. A *S. aureus* infected murine model of surgical excision wound showed a significant reduction in MPO expression at the wound site after treatment with a novel hyaluronic acid-binding peptide, while also indicating a reduction in bacterial burden [49]. Anti-inflammatory role of *V. polyanthes* Less. in croton-oil induced ear edema of Swiss Albino mice showed down regulation of MPO, simultaneously reducing inflammatory cell infiltration [50].

**Fig. 6:**
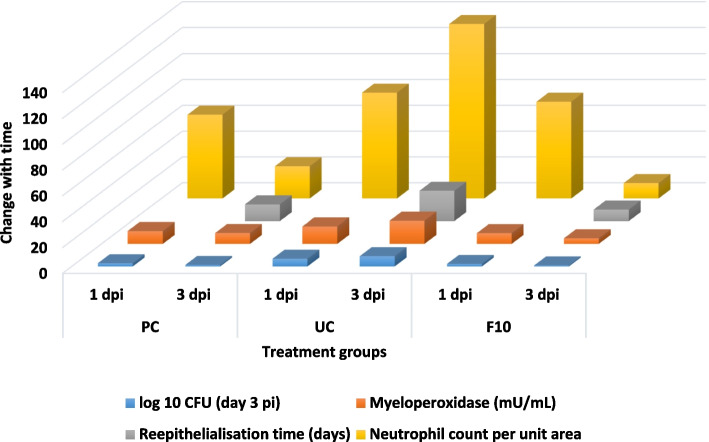
Correlation of three parameters in zebrafish infected wound tissue upon treatment of with *V. arborea* F10 (0.5%). Log_10_ CFU (for *E. coli* for example), neutrophil population and myeloperoxidase enzyme activity on 1 and 3 dpi and reepithelialisation time for the PC, UC and F10 groups are indicated. PC, positive control, UC, untreated control. All parameters were found to increase with the UC

MPO, measured as a marker of polymicrobial infection, correlated to the reduction in microbial load and neutrophil profile (Figs. 2, 4, 6 and 10) in F10 treated wounds which is the first report in adult Zebrafish. In the untreated control all three parameters, viz. bacterial populations, neutrophil count and MPO increased.

### Kinetics of immuno-molecular markers

**Fig. 7:**
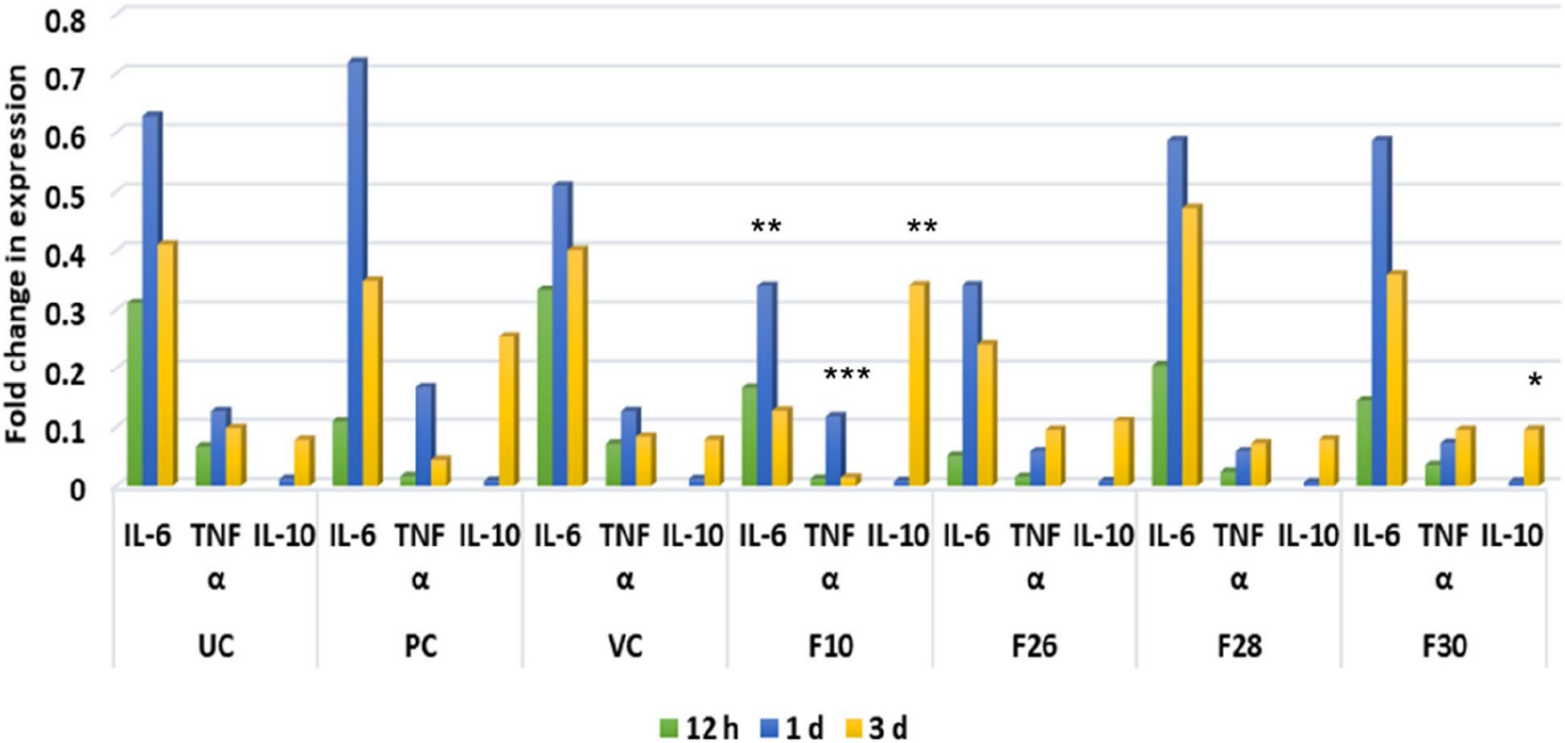
Fold change in expression of inflammatory cytokines. The change across the control and treated zebrafish (0.5%) groups (*V. arborea* F10, F26, F28, F30 fractions) over time, days post infection (dpi) showed decrease of pro-inflammatory cytokines- IL-6 and TNF-α upon treatment. Increased expression of anti-inflammatory cytokine IL-10 from day1 to day 3 in the treatment groups was noted. Values are mean [*n* = 20] ±SD, *p < 0.05, **p < 0.01 compared to positive or untreated control, respectively. UC, untreated control; PC, positive control; VC, vehicle control

Neutrophils modulate inflammation also by producing different pro- and anti-inflammatory cytokines that essentially attract more immune cells to the wound site [51]. The anti-bacterial fractions of *V. arborea* in the study, modulated the expression of pro- and anti-inflammatory cytokines, reducing the former and upregulating the latter (Fig. 7), contributing to better resolution of inflammation.

The tissue samples of infected wound, and treated (0.5% ointment) tissue were used for expression profiles of the cytokines during inflammatory phase. Treatment with test fractions modulated expression of IL-6, TNF-α and IL-10 compared to the untreated group. The expression of pro-inflammatory cytokines IL-6 and TNF-α reduced upto 18 and 7.8 fold from 1 to 3 dpi. The expression of anti-inflammatory cytokine IL-10 increased 33 folds from 1 to 3 dpi (Fig. 7). On balance, the inflammatory cytokine profile post treatment with F10 (0.5%) indicated scope for better and faster healing of the infected wound tissue.

*V. amygdalina* leaf extracts possessed modulating role in altering expression of IL-6 and IL-10 during *S. aureus* infection. IL-6 deficiency is understood to result in impaired cutaneous wound healing, studied in immunosuppressed mice by Gallucci et al. [52]. However, prolonged expression of IL-6 is understood to trigger chronic inflammation and delayed healing by stimulating T- and B- cells [53]. According to [54], increased expression of TNF-α in wound tissue, beyond the inflammatory phase, results in impaired cutaneous wound healing both in chronic and impaired acute wounds (such as wounds with infection). Thus, the balanced and time-bound expression of these pro-inflammatory mediators is essential for regulated inflammation, which prevents transition of the wound from acute to chronic state. The data presented here shows for the first time that F10 at 0.5% balanced the pro- and anti-inflammatory cytokines and promoted healing of infected wound tissues.

### Wound repair kinetics

#### Nitrite level in infected wound tissue

Nitric oxide, a diffusible intercellular signalling molecule is generated by the inducible nitric oxide synthase (iNOS), found in the macrophages [55]. Nitric oxide (NO) has a potent role in wound healing, modulating the chemoattractant cytokines, and thus regulating post-wound inflammation, keratinocyte migration, proliferation and tissue remodelling. The level of NO in wound tissue is an indication of healing and repair [56].

**Fig. 8:**
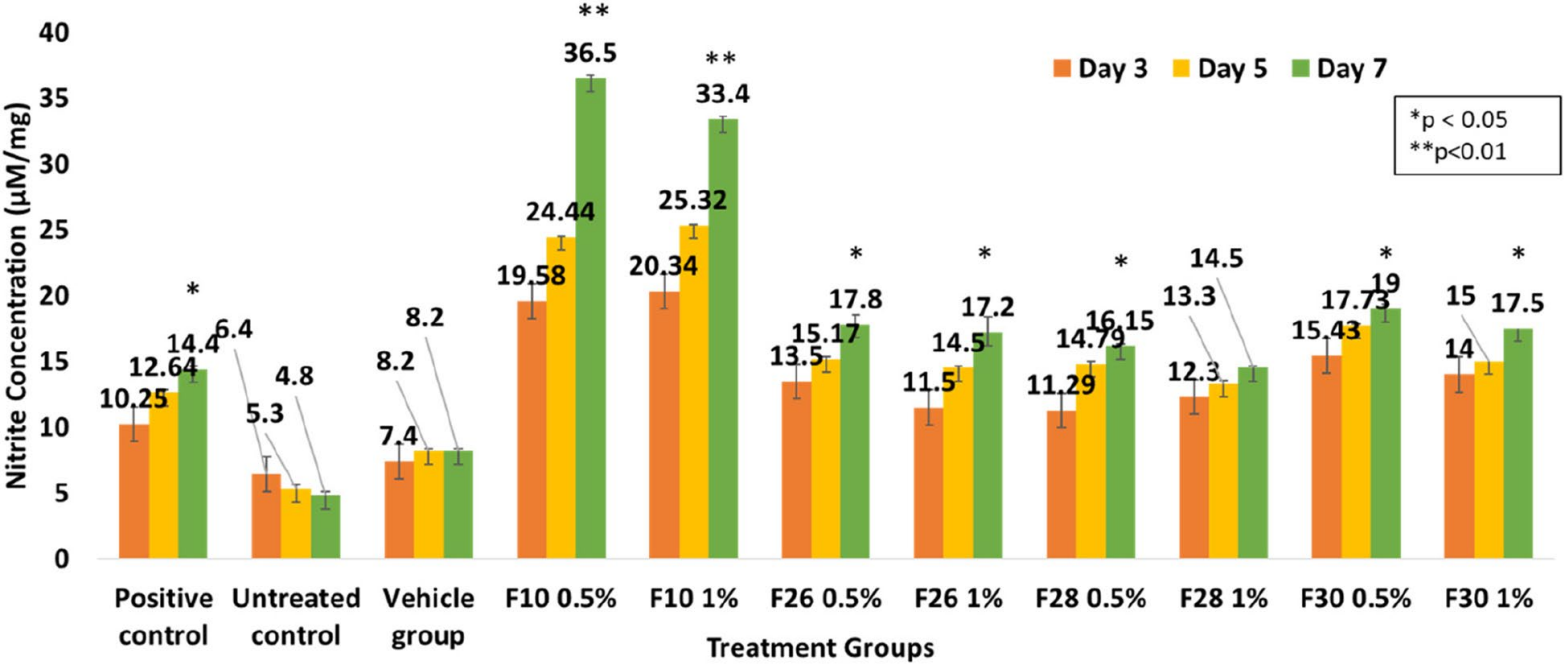
Nitrite level in zebrafish infected wound tissue on 3, 5 and 7 dpi. *V. arborea* bioactive fractions show optimal increase in nitrite concentration compared to controls. Values are mean [*n* = 20] ±SD, *p < 0.05, **p < 0.01 compared to positive or untreated control, respectively

Treatment of the infected wound tissue with test fractions at 0.5 & 1% increased nitrite levels during the tissue remodelling phase of healing. F10 increased the level by 2.53 fold more than the positive control group 7dpi (Fig. 8).

Nitrite levels in the wound lysate correlated with healing in several previous reports. In experimentally diabetic male Sprague–Dawley rats, nearly 14-fold less nitrite concentration in the wound lysate was observed compared to the normal control, indicating delayed wound healing and incomplete collagen deposition, the condition was restored partially by insulin treatment [57]. Nitrite therapy proved to be effective in one of the earlier reports where patients with methicillin-resistant *S. aureus* (MRSA) infected wounds were considered. There was a reduction in infection and acceleration of healing with application of topical acidified nitrite [58].

#### Upregulation of iNOS accelerates wound repair in infection-impaired wounds

**Fig. 9:**
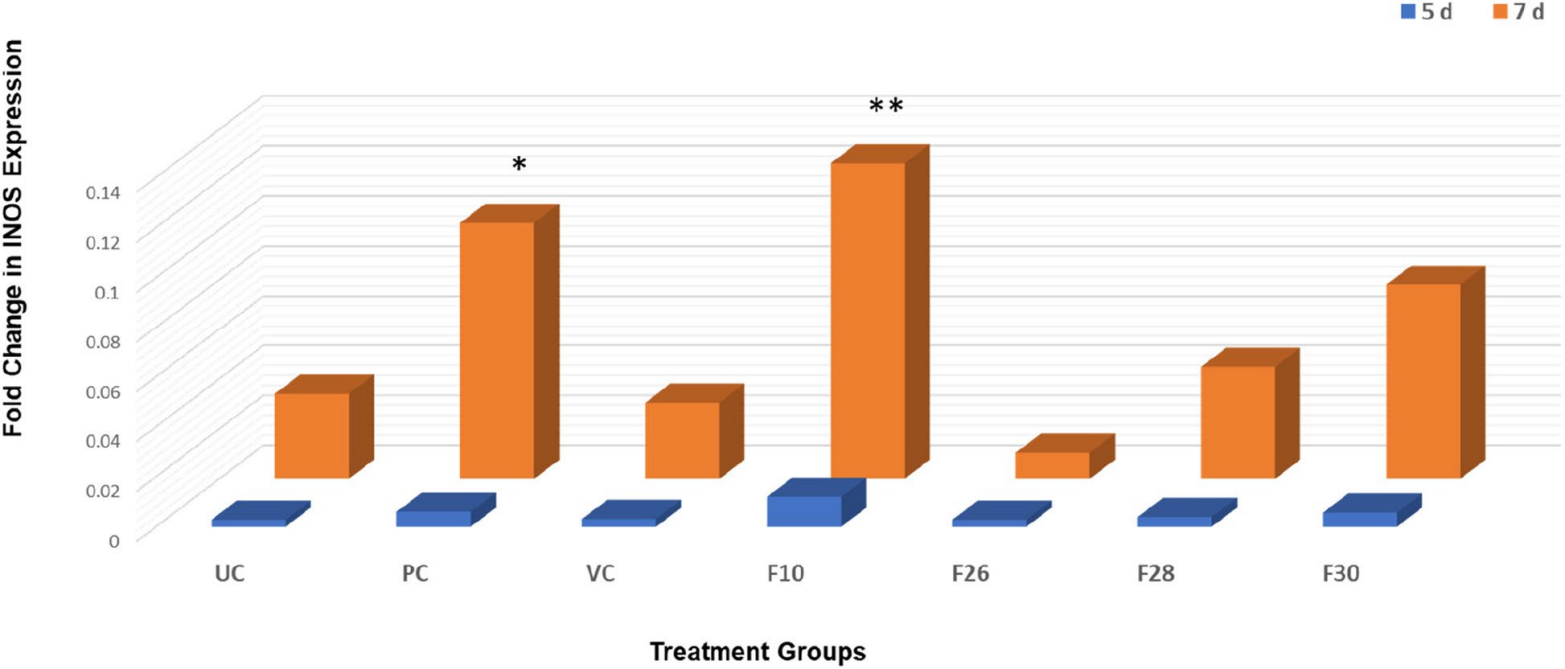
Fold change in expression of iNOS in zebrafish control and treatment (0.5% *V. arborea* fractions) groups over time, days post infection (dpi). Values are mean [*n* = 20]± SD, *p < 0.05, **p < 0.01 compared to positive or untreated control, respectively. UC, untreated control; PC, positive control; VC, vehicle control

Enhanced nitrite levels favoured cellular signalling for tissue remodelling in infected wound tissue and is generated by the Inducible nitric oxide synthase iNOS; [36, 58]. Since 0.5% ointments were effective in inducing high nitrite levels (Fig. 6) in the wounded, infected tissues, only this concentration was evaluated for iNOS expression. Treatment with test fractions modulated expression of iNOS compared to the untreated group. The expression of iNOS in F10 treated group was 1.2 fold higher than the positive control and there was a tenfold increase from 5 to 7 dpi in the F10 treated group (Fig. 9). These results correlated with the nitrite levels in the infected wound tissue from 5 to 7 dpi (Fig. 8).

A report from Stallmeyer et al. [36] showed that the experimental inhibition of iNOS by L-N^6^-(1-iminoethyl) lysine, a selective inhibitor of iNOS, resulted in a straggled keratinocyte reepithelialisation. The study also correlated the iNOS inhibition and the low nitrite levels in the wound lysate. Dermal fibrobalsts derived from the iNOS deficient experimental mice did not proliferate efficiently and produced very less collagen when grown in the presence of fetal bovine serum. Addition of nitric oxide (NO) to the fibroblast cultures resulted in good response in proliferation and collagen synthesis. The collagen lattice contraction was several times better after the NO supplementation [59]. Phytocompounds from *Sideroxylon obtusifolium*, a Brazilian tree, has been reported to enhance wound healing in surgical dorsal skin wound model of male Swiss mice, by inducing 53% increase in iNOS production. This supported collagen formation and reepithelialisation of the wounded tissue [60].

#### Reepithelialisation of infected wounds

**Fig. 10:**
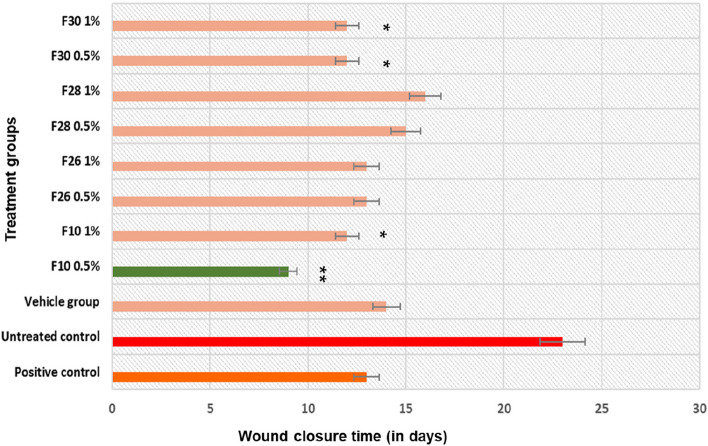
Wound closure time observed across the zebrafish wound models with infection, treated with *V. arborea*. Values are mean [*n* = 20] ±SD, *p < 0.05, **p < 0.01 compared to positive or untreated control, respectively

The time or days required for reepithelization was observed in the infected and treated wounds. The untreated, infected wounds showed complete reepithelialisation by 23 dpi. Impairment due to infection delayed healing by 6 days. The treatment with positive control and the bioactive fractions enhanced healing and reduced the reepithelialisation time comparable to the healing of acute cutaneous wounds. Treatment with F10 showed reepithelialisation around 9 dpi (Fig. 10 and wound contraction, Fig. S4). F26 and F30 showed reepithelialisation by 12 dpi. F28 treatment showed similar but slightly delayed reepithelialisation time around 16 dpi (Fig. 10). Wound closure (mm) was threefold better in treated groups compared to untreated ones as shown in supplementary Table S5.

The biofilms of *Staphylococcus* were shown to delay reepithelialisation in murine cutaneous wound model. The study by Schierle et al. [61] demonstrated the effect of RNAIII inhibiting peptide in reducing Staphylococcal bioburden thereby reducing reepithelialisation time by 2 days.

Methanolic leaf extracts of *Elaeis guineensis* reduced reepithelization time in *S. aureus* infected Sprague Dawley rat model of full thickness wound. The extract treated wounds showed 100% wound closure by day 16 while the untreated control group showed 65% wound closure by day 16. The extract reduced CFU to 0 by day 16 which was not seen in the untreated control [62]. The result of the current study is similar to the previous reports on reepithelialisation of infected wounds.

The healing of a cutaneous excision wound requires an initial anti-inflammatory activity for resolution of inflammation and enhanced NO signals. The increase in NO accelerated keratinocyte migration, collagen synthesis and matrix formation for better wound reepithelialisation and repair under impaired conditions like the wound infection [63, 64]. Fraction 10 at 0.5% w/w showed potent anti-microbial property ex-vivo, accelerated wound healing and facilitated repair. The adult Zebrafish mechanical cutaneous wound infection model established, supports the efficacy of the bioactive fractions in the current study as wound healing agents that modulate multifaceted cellular and gene expression profiles favourably rather than possessing a single holistic role.

## Conclusion

**Fig. 11:**
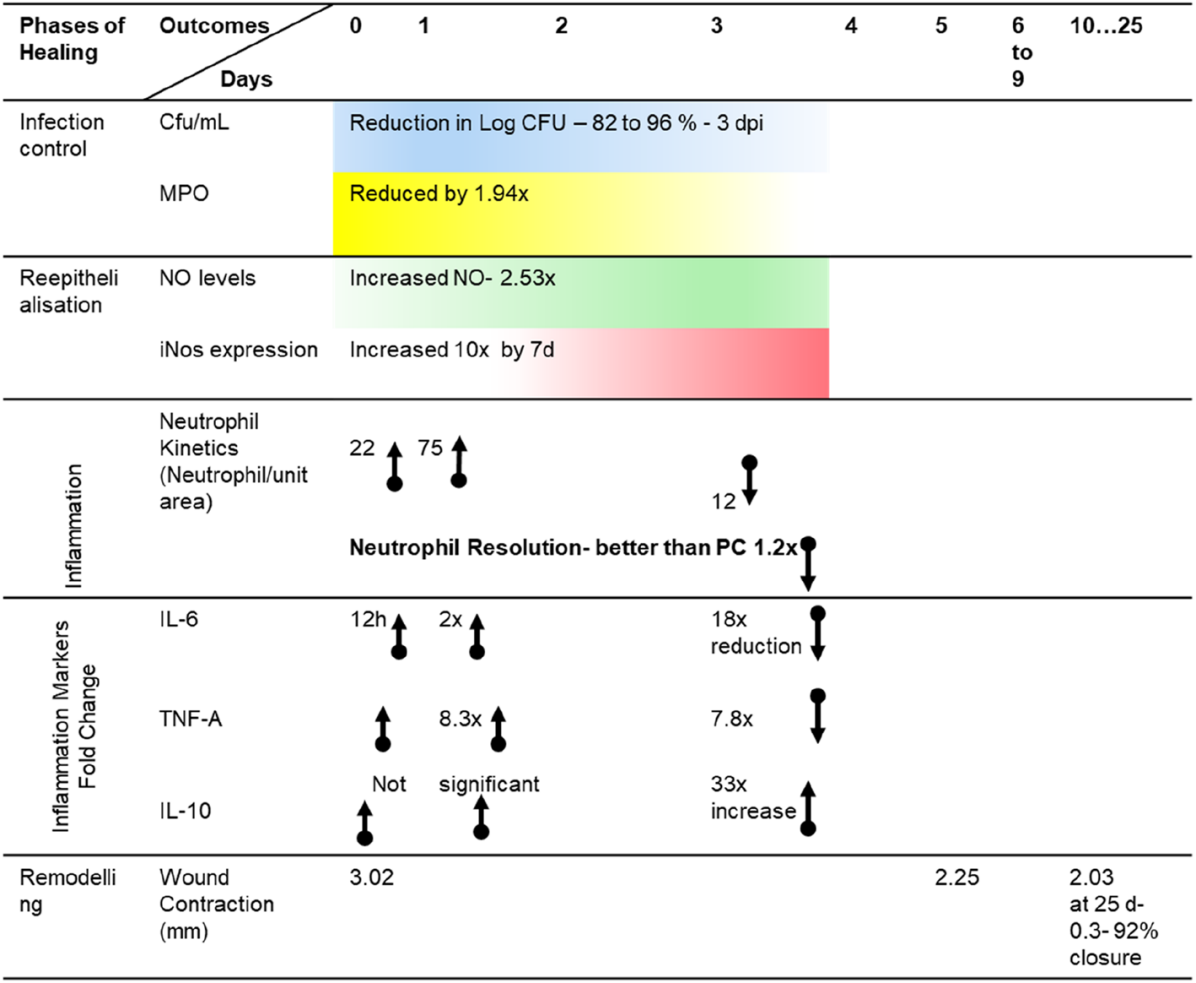
Healing model of Infected Wounds using *V.* *arborea* extracts in Adult Zebrafish –Kinetics using visual, CFU reduction, histopathology, cytology, biochemical and immune-molecular markers at different phases

The zebrafish wound infection model evaluated here, mimics mammalian impaired wound microenvironment, proportionate to the complexity of the species, that makes it possible to screen phytoextracts for wound healing potency. The cellular interplay, modulation of inflammation, destruction of microbes at the wound site, undulating pattern of inflammatory cytokines, positive regulation of cell migration, were shown to be possible in the developed model. Owing to the *e*x-vivo anti-microbial activity and potency to accelerate wound healing under impaired conditions, Fraction 10 from the hexane leaf extract of *V. arborea* at 0.5% concentration could be evaluated for pre-clinical and clinical therapeutic applications. The Fig. 11 summarises the kinetics measurable during the interplay of phases in wound healing based on our observations.

### Supplementary Information

**Additional file 1: Fig. S1.** Time kill kinetics of *V. arborea* Fraction 26 against all five test strains with the respective MIC and AUC of log CFU shown adjacent. UC, untreated control; PC, positive control (ampicillin at inhibitory concentration); *n* = 3; values are mean ± SEM. ** *p*<0.05, *** *p*<0.01 compared to the untreated control (one-way ANOVA followed by Dunnett’s post hoc test). **Fig. S2.** Time kill kinetics of *V. arborea* Fraction 28 against all five test strains with the respective MICand AUC of log CFU shown adjacent. UC, untreated control; PC, positive control (ampicillin at inhibitory concentration); *n* = 3; values are mean ± SEM. ** *p*<0.05, *** *p*<0.01 compared to the untreated control (one-way ANOVA followed by Dunnett’s post hoc test). **Fig. S3.** Time kill kinetics of *V. arborea* Fraction 30 against all five test strains with the respective MICand AUC of log CFU shown adjacent. UC, untreated control; PC, positive control (ampicillin at inhibitory concentration); *n* = 3; values are mean ± SEM. ** *p*<0.05, *** *p*<0.01 compared to the untreated control (one-way ANOVA followed by Dunnett’s post hoc test). **Fig. S4.** Wound contraction observed in the adult zebrafish acute cutaneous wound model on 0, 5, 7 and 10 dpw represented by treatment with 0.5% F10 fraction of *V. arborea*. pc, positive control (0.5% povidone iodine ointment treated); ut, untreated control; vc vehicle control. Scale bar, 3 mm. **Table S1.** Biological activities of extracts of various species of *Vernonia*. **Table S2.** Biological activities of compounds isolated from various species of *Vernonia*. **Table S3.** Anti-microbial activity of *Vernonia sp*. against Gram positive and Gram negative bacteria. **Table S4.** Quantification of phytoconstituents in the hexane leaf extracts of *V. arborea*. **Table S5.** Wound closure of infected tissues observed in adult zebrafish treated with 0.5% *V. arborea* fractions and control groups. Wound closure (WC) was 3-fold better in treated groups compared to untreated ones. Values were significant with *p*<0.05.

## Data Availability

All data generated or analysed during this study are included in this published article [and its supplementary information files].

## Abbreviations

IL: Interleukin
TNF: Tumor Necrosis Factor
ATCC: American Type Culture Collection
CFU: Colony Forming Unit
REMA: Resazurin Microtitre Assay
MIC: Minimum Inhibitory Concentration
OD: Optical Density
H&E: Haematoxylin & Eosin
AUC: Area Under the Curve
MPO: Myeloperoxidase
iNOS: Inducible Nitric Oxide Synthase
NO: Nitric Oxide
MRSA: Methicillin Resistant *Staphylococcus aureus*
