# Supplementary material for: Anti-bacterial and anti-inflammatory properties of Vernonia arborea accelerate the healing of infected wounds in adult Zebrafish
Source: BMC Complement Med Ther. 2024 Feb 19;24:95. doi: 10.1186/s12906-024-04383-8 (PMC10875872; doi:10.1186/s12906-024-04383-8)
Supplement: Supplementary file 1 — Additional file 1: Fig. S1. Time kill kinetics of V. arborea Fraction 26 against all five test strains with the respective MIC and AUC of log CFU shown adjacent. UC, untreated control; PC, positive control (ampicillin at inhibitory concentration); n = 3; values are mean ± SEM. ** p<0.05, *** p<0.01 compared to the untreated control (one-way ANOVA followed by Dunnett’s post hoc test). Fig. S2. Time kill kinetics of V. arborea Fraction 28 against all five test strains with the respective MICand AUC of log CFU shown adjacent. UC, untreated control; PC, positive control (ampicillin at inhibitory concentration); n = 3; values are mean ± SEM. ** p<0.05, *** p<0.01 compared to the untreated control (one-way ANOVA followed by Dunnett’s post hoc test). Fig. S3. Time kill kinetics of V. arborea Fraction 30 against all five test strains with the respective MICand AUC of log CFU shown adjacent. UC, untreated control; PC, positive control (ampicillin at inhibitory concentration); n = 3; values are mean ± SEM. ** p<0.05, *** p<0.01 compared to the untreated control (one-way ANOVA followed by Dunnett’s post hoc test). Fig. S4. Wound contraction observed in the adult zebrafish acute cutaneous wound model on 0, 5, 7 and 10 dpw represented by treatment with 0.5% F10 fraction of V. arborea. pc, positive control (0.5% povidone iodine ointment treated); ut, untreated control; vc vehicle control. Scale bar, 3 mm. Table S1. Biological activities of extracts of various species of Vernonia. Table S2. Biological activities of compounds isolated from various species of Vernonia. Table S3. Anti-microbial activity of Vernonia sp. against Gram positive and Gram negative bacteria. Table S4. Quantification of phytoconstituents in the hexane leaf extracts of V. arborea. Table S5. Wound closure of infected tissues observed in adult zebrafish treated with 0.5% V. arborea fractions and control groups. Wound closure (WC) was 3-fold better in treated groups compared to untreated ones. Values were signif [file 12906_2024_4383_MOESM1_ESM.docx]

**Supplementary data**


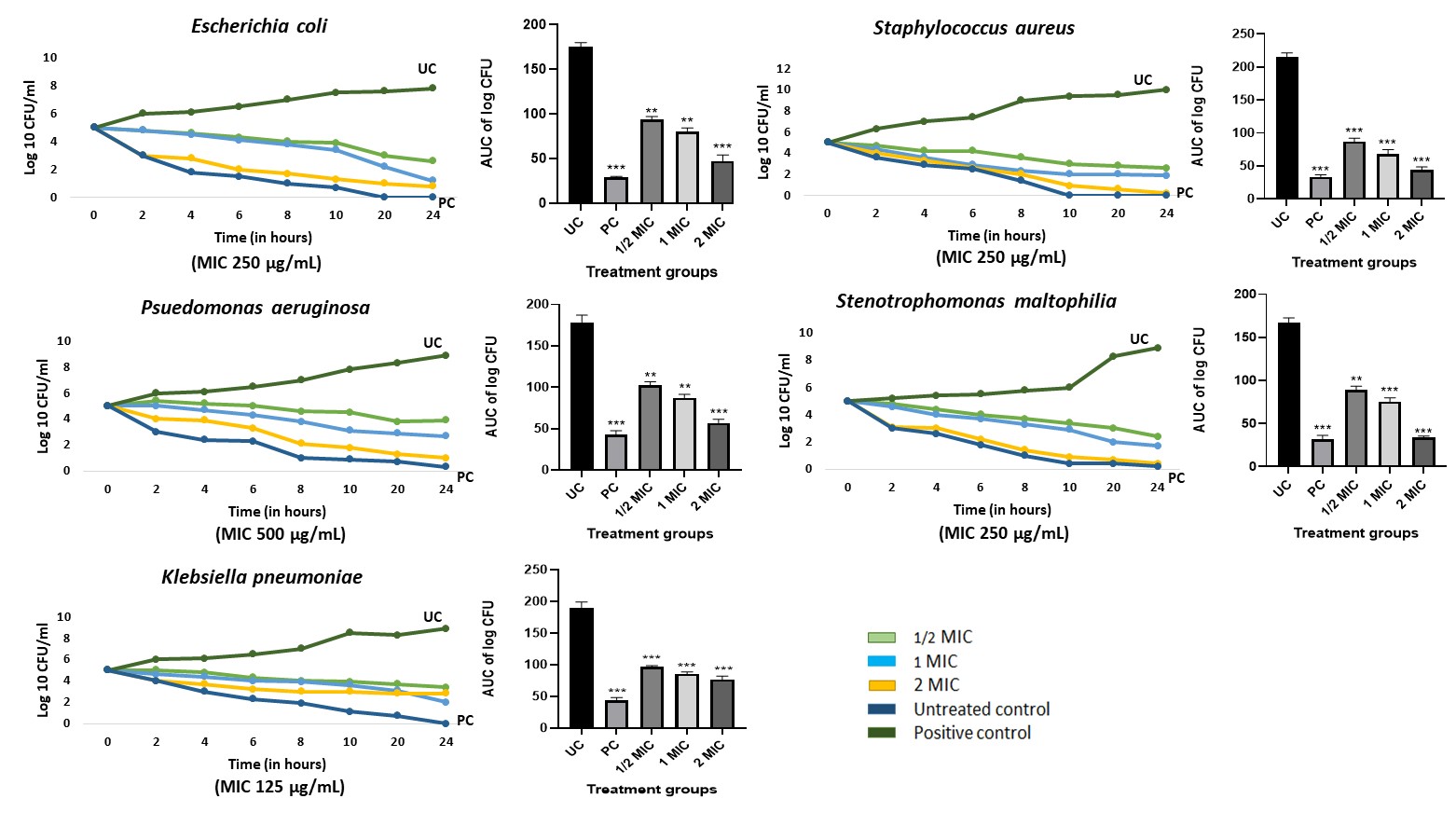


**Fig. S1. Time kill kinetics of *V. arborea* Fraction 26 against all five test strains with the respective MIC and AUC of log CFU shown adjacent.** UC, untreated control; PC, positive control (ampicillin at inhibitory concentration); n = 3; values are mean ± SEM. ** p<0.05, *** p<0.01 compared to the untreated control (one-way ANOVA followed by Dunnett’s post hoc test)


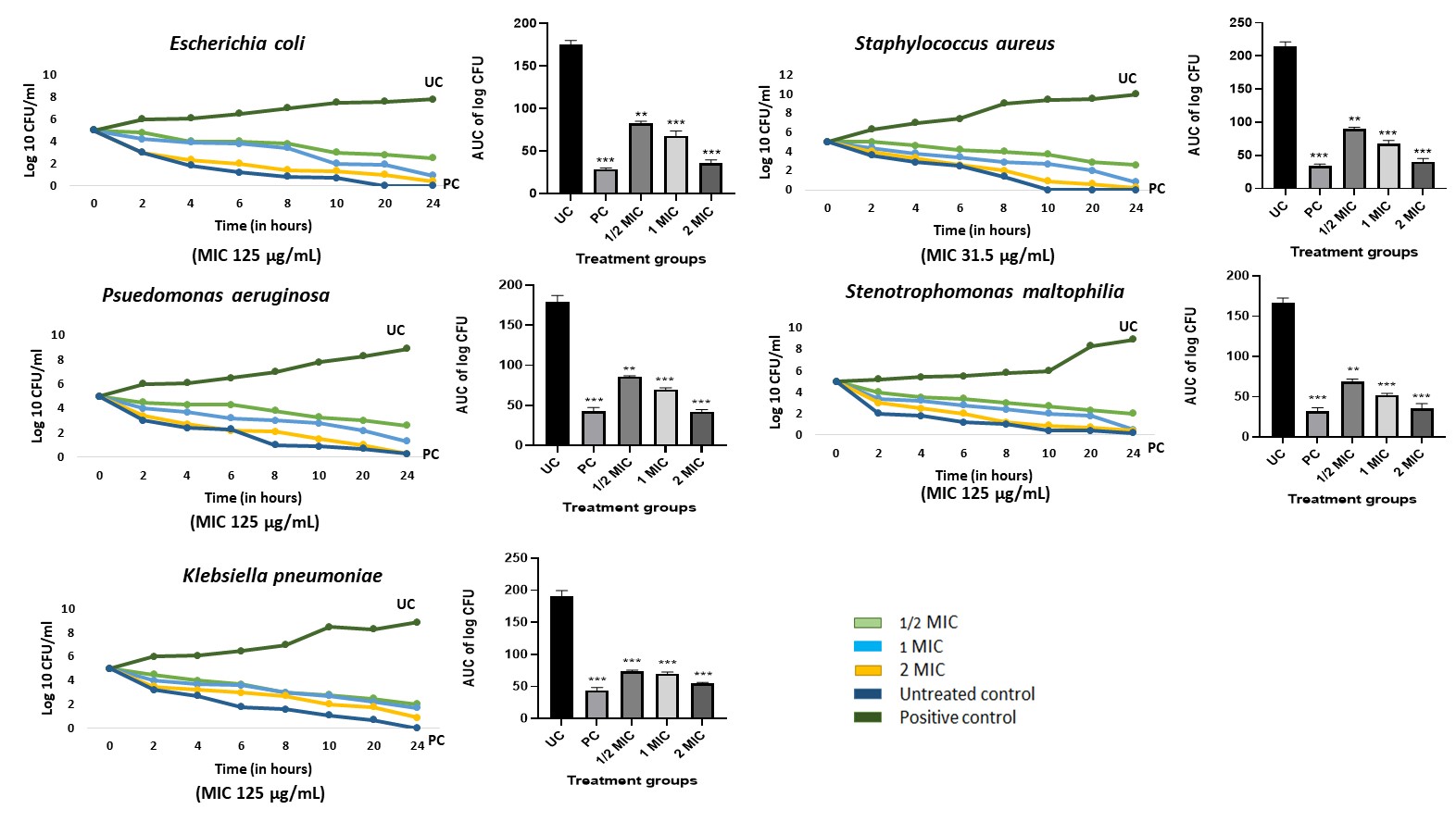


**Fig. S2 Time kill kinetics of *V. arborea* Fraction 28 against all five test strains with the respective MICand AUC of log CFU shown adjacent.** UC, untreated control; PC, positive control (ampicillin at inhibitory concentration); n = 3; values are mean ± SEM. ** p<0.05, *** p<0.01 compared to the untreated control (one-way ANOVA followed by Dunnett’s post hoc test)


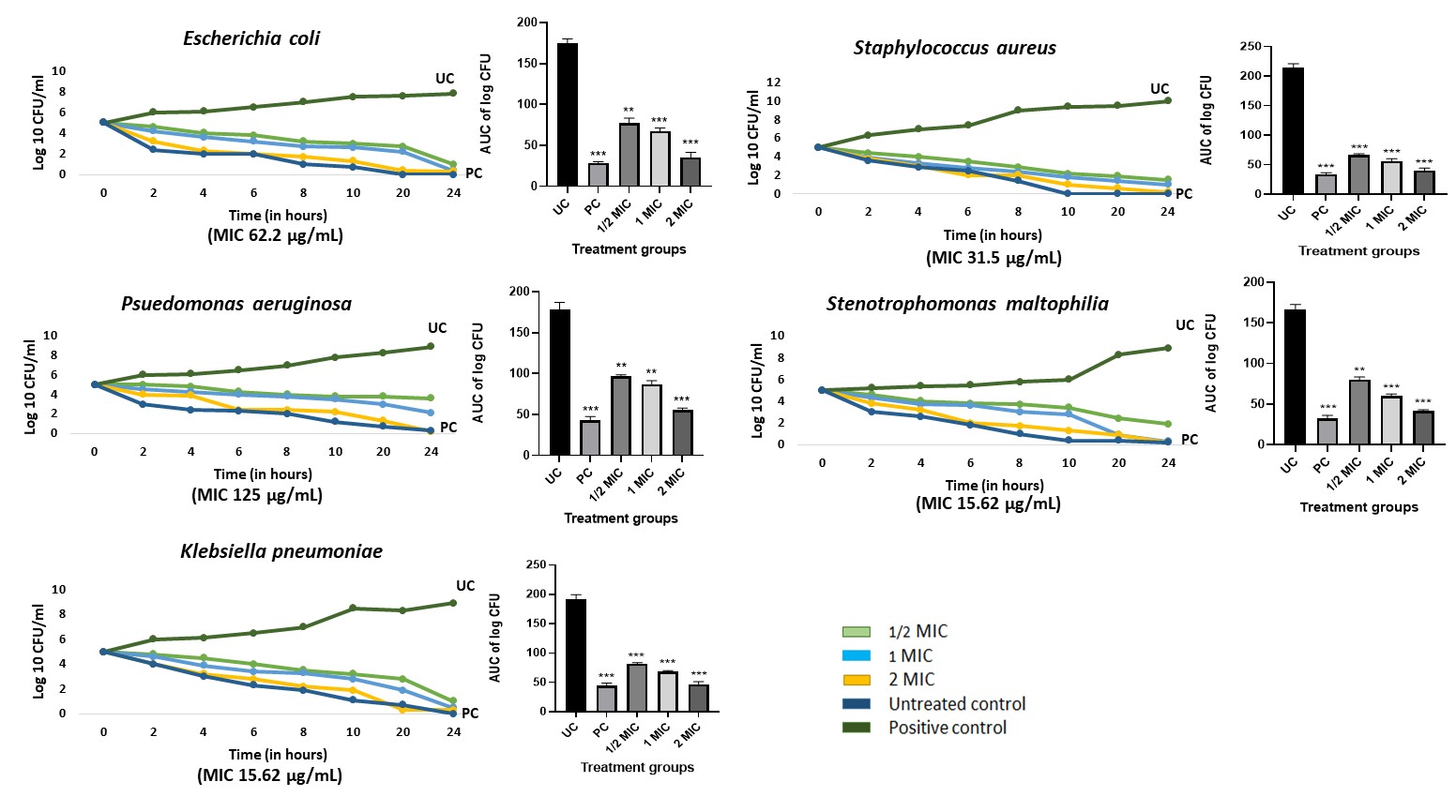


**Fig. S3 Time kill kinetics of *V. arborea* Fraction 30 against all five test strains with the respective MIC and AUC of log CFU shown adjacent.** UC, untreated control; PC, positive control (ampicillin at inhibitory concentration); n = 3; values are mean ± SEM. ** p<0.05, *** p<0.01 compared to the untreated control (one-way ANOVA followed by Dunnett’s post hoc test)


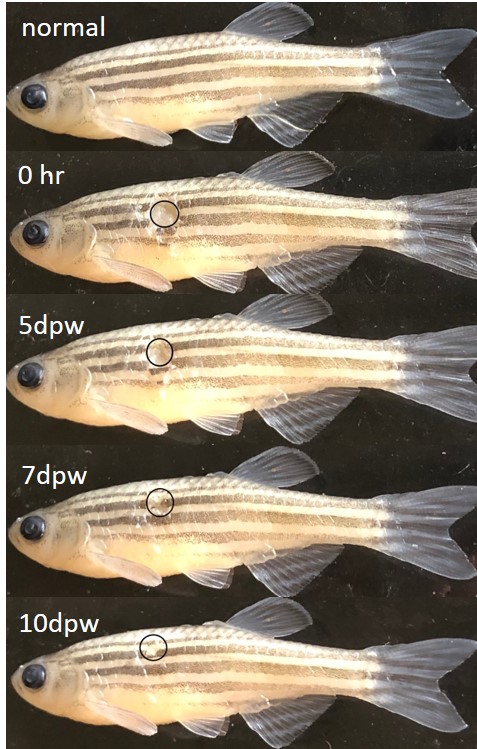

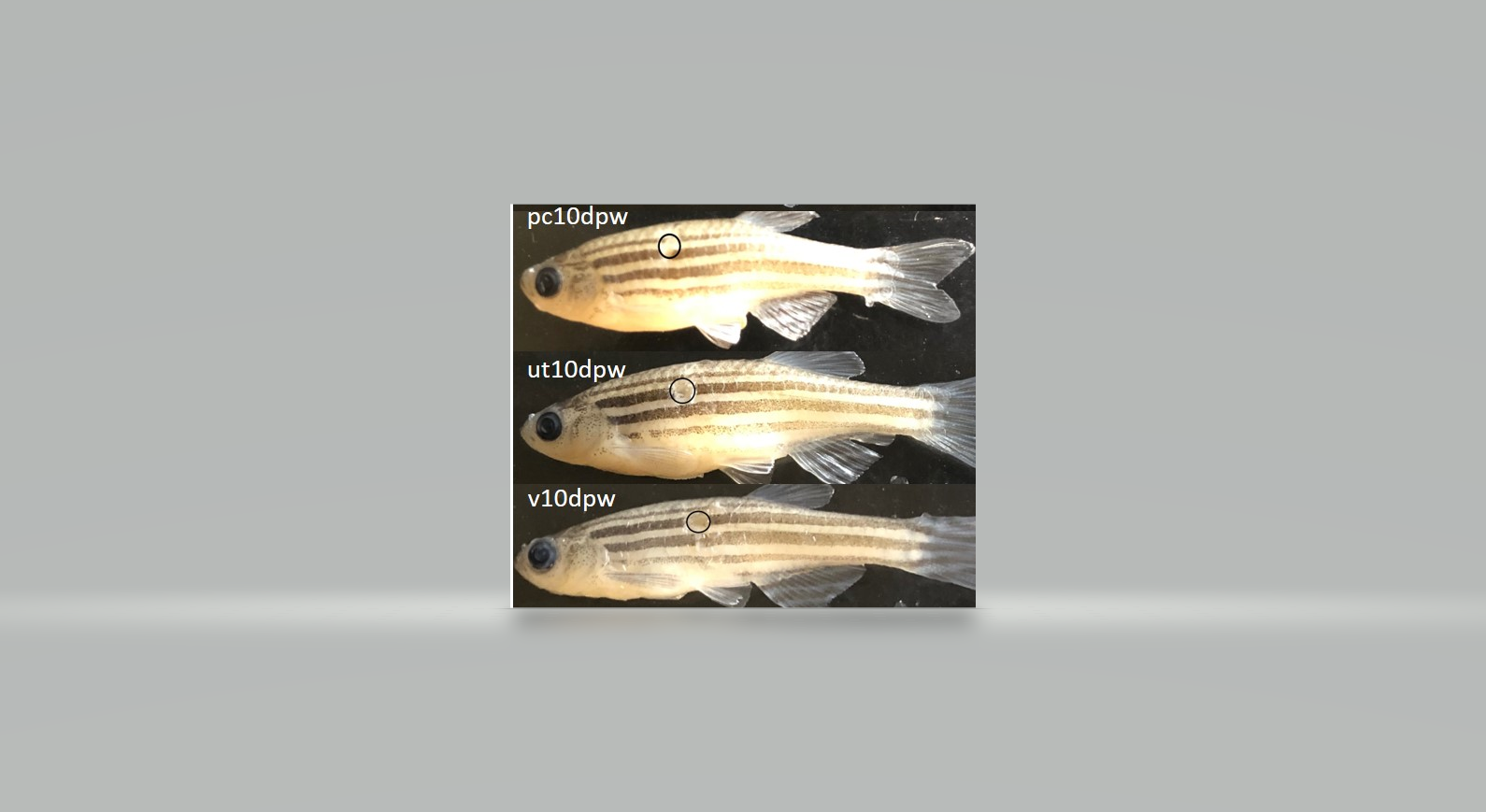

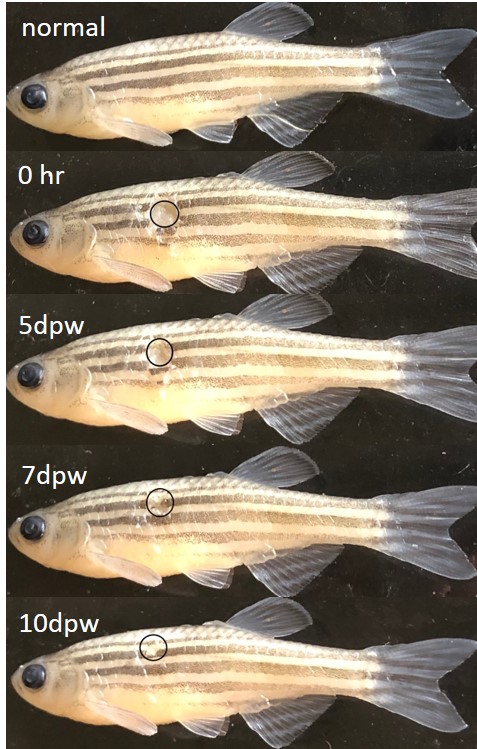

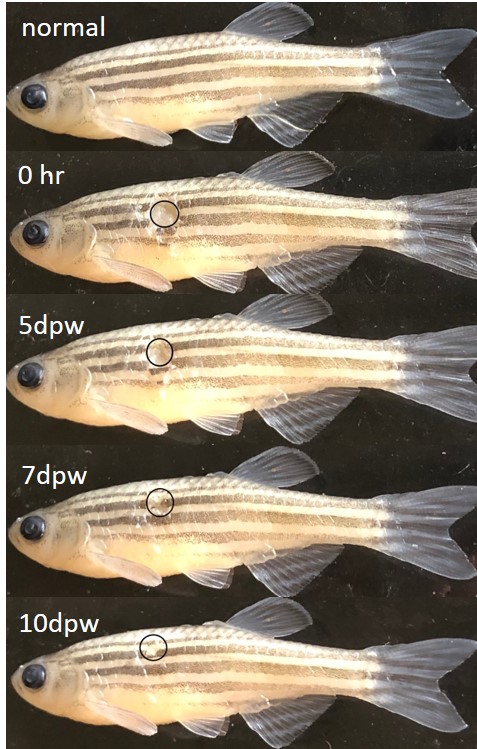

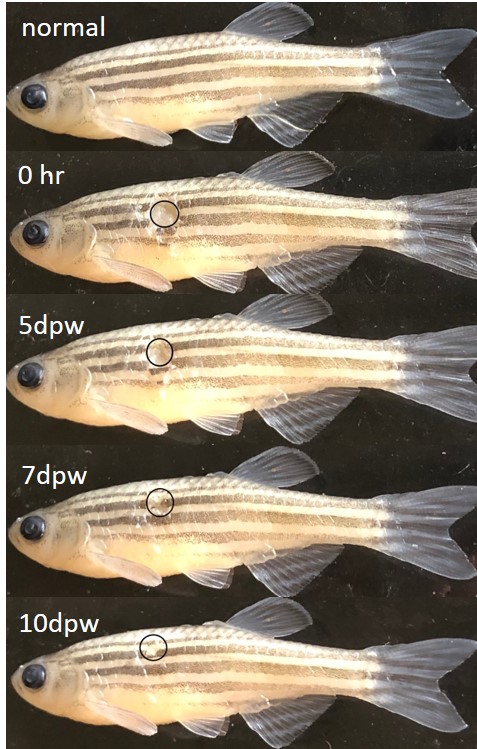

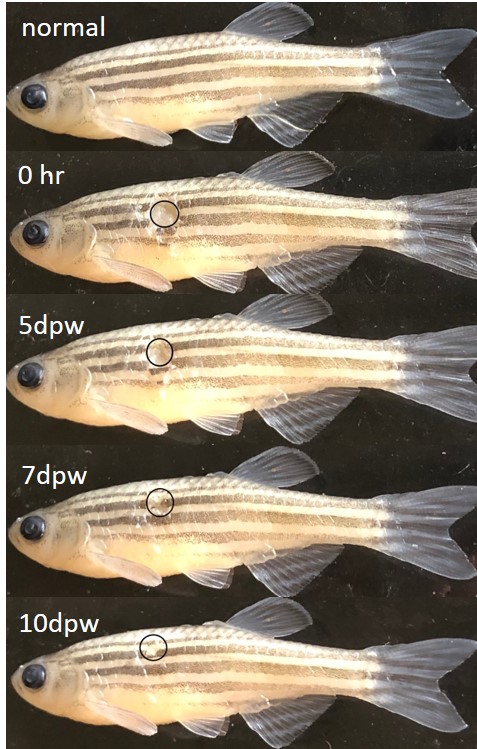


**0hpw**

**5dpw**

**7dpw**

**10dpw**

**Fig. S4 Wound contraction observed in the adult zebrafish acute cutaneous wound model on 0, 5, 7 and 10 dpw represented by treatment with 0.5% F10 fraction of *V. arborea*.** pc, positive control (0.5% povidone iodine ointment treated); ut, untreated control; vc vehicle control. Scale bar, 3 mm

**Supplementary Tables**

**Table S1: Biological activities of extracts of various species of *Vernonia***

| **S. No.** | **Species reported** | **Plant part** | **Type of extract** | **Activity reported** | **Bioactive Concentration/Quantitative measure of potency** | **Reference** |
| --- | --- | --- | --- | --- | --- | --- |
| 1 | *Vernonia amygdalina* | Leaf | Water | Anti-dermatophytic activity | *Epidermophyton*: 58.60 mg/ml; *Microsporum*: 49.40 mg/ml | Iliyasu et al., 2019 |
|  |  |  |  |  | *Trichophyton*:55.80 mg/ml; Mixed growth 56.10 mg/ml |  |
|  |  |  | Ethanol |  | *Epidermophyton*: 65.10 mg/ml; *Microsporum*: 57.50 mg/ml |  |
|  |  |  |  |  | *Trichophyton*: 58.20 mg/ml; Mixed growth 62.30 mg/ml |  |
| 2 | *Vernonia amygdalina* | Leaf | Ethanol | Anti-fungal activity  (Zone of inhibition) | *Trichophyton tonsurans*:16 ± 0.02 mm | Iyanuloluwa et al., 2019 |
|  |  |  |  |  | *Trichophyton rubrum*: 14 ± 0.02 mm (Zone of inhibition) |  |
| 3 | *Vernonia polysphaera* | Leaf | Ethanol | Anti-microbial | 13-20 mm (zone of inhibition against S. aureus) | da Cruz et al., 2019 |
|  |  |  |  |  | 3.12 - 12.5 mg/mL (MIC against S. aureus) |  |
| 4 | *Vernonia condensata* | Leaf | Water | Cytotoxic activity in cancer cell lines | 26.0 mg/ml (IC 50 at 48 hrs) | Thomas et al., 2016 |
|  |  |  |  | Leukemia | 10 mg/ml (IC 50 at 48 hrs) |  |
|  |  |  |  | Breast adenocarcinoma | 11.9 mg/ml (IC 50 at 48 hrs) |  |
| 5 | *Vernonia amygdalina* | Root | Ethanol | Anti-leukemic activity | 20 µg/mL (86% inhibition of ALL cells) | Khalafalla et al., 2009 |
| 6 | *Vernonia cinerea* | Leaf | Water | Anti-hyperglycaemic activity | 300 mg/kg in alloxan-induced diabetic rats | Choudary et al., 2013 |
| 7 | *Vernonia amygdalina* | Leaf | Ethanol | Anti-diabetic activity | 0.51 mg/mL (IC50 value – in vitro enzyme inhibitory assay) | Alara and Abdurahman, 2019 |
| 8 | *Vernonia arborea* | Leaf | Methanol | **Topical Wound healing activity** | 5% w/w ointment in Wistar rats | Manjunatha et al., 2005 |
|  |  |  |  |  | 30 mg/kg bw oral administration dose |  |
| 9 | *Vernonia arborea* | Leaf | Hexane | *In-vitro* antimicrobial activity against five selected wound pathogens | MIC ranging from 31.5 µg/mL against *Staphylococcus aureus* and *Klebsiella pneumoniae* to 125 µg/mL against *Pseudomonas aeruginosa*; least MBC was 31.5 µg/mL against *S. aureus* and K. *pneumoniae* | Vaidyanathan and Lokeswari, 2021 |
| 10 | *Vernonia arborea* | Leaf | Hexane | **Acute cutaneous wound healing activity** | 0.5% and 1% ointment in adult Zebrafish  3-fold increase in neutrophil count on 1 dpw; 1.6 times better resolution of inflammation; 15.48% increase in reduced glutathione; 3-fold reduction in malondialdehyde; 2-fold increase in tissue markers like hydroxyproline, hexosamine and hexuronic acid; 92.3% wound contraction | Vaidyanathan and Lokeswari, 2022 |
| 11 | *Vernonia scorpioides* | Leaf | Ethanol | **Topical Wound healing activity** | 200 mg topical hydrogel in guinea pigs | Leite et al., 2002 |
| 12 | *Vernonia amygdalina* | Leaf | Water | **Wound healing activity** | 20 µg oral administration in rat excision wound models | Nafiu et al., 2016 |

**Table S2: Biological activities of compounds isolated from various species of *Vernonia***

| **S. No.** | **Species reported** | **Plant part** | **Type of extract** | **Compound isolated** | **Activity reported** | **Bioactive Concentration/Quantitative measure of potency** | **Reference** | **Reported yield of the bioactive compound** | **Calculated % yield** |
| --- | --- | --- | --- | --- | --- | --- | --- | --- | --- |
| 1 | *Vernonia amygdalina* | Leaf | Chloroform | Vernodalin | Cytotoxic activity against human carcinoma of nasopharynx | 18 µg/ml | Kupchan et al., 1969 | 60 mg/400 g crude | 0.02% |
|  |  |  |  | Vernomygdin |  | 15 µg/ml |  | 30 mg/400 g crude | 0.01% |
| 2 | *Vernonia amygdalina* | Leaf | Methanolic | Vernolide | Anti-plasmodium activity | 18.1 µg/ml | Abhay et al., 2015 | 125.3 mg/30 g crude | 0.42% |
|  |  |  | Ethanolic | Vernodalol |  | 16.1 - 21.7 µM |  | 108.5 mg/30 g crude | 0.36% |
| 3 | *Vernonia amygdalina* | Flower | Acetone | Vernolide | Anti-bacterial activity- Agar Diffusion | S*. aureus*: 19 mm; B. *subtilis*: 12 mm; E. *coli*:10 mm; K. *pneumonia*: 12 mm; P. *mirablis*: 6 mm | Habtamu and Melaku, 2018 |  |  |
|  | *Vernonia amygdalina* | Flower | Acetone | Isorhamnetin |  | S. *aureus*: 11 mm; B. *subtilis*: 9 mm; E. *coli*:10 mm; K. *pneumonia*: 14 mm; P. *mirablis*: 12 mm |  |  |  |
| 4 | *Vernonia*  *adoensis* | Leaf | Acetone | Chondrillasterol | Anti-bacterial activity- MIC: | P. *aeruginosa*: 1.6 ug/ml | Mozirandi, 2017 |  |  |
| 5 | *Vernonia galamensis* | Leaf | Acetone | Vernolide | Anti-bacterial activity: Agar Diffusion | 17.7±1.5 (S. *aureus*); 18±1.0 (S. *boydii*); 18.7±0.6 (S. *typhii*) | Tafesse et al., 2018 | 1.6 mg/g crude | 0.16% |
|  |  |  |  |  | MIC- 20 mg/ml | S. *aureus;* S. *boydii*; S. *typhii* |  |  |  |
|  |  |  |  | Vernonioside | Anti-bacterial activity: Agar Diffusion | 28.7±1.5 (S. *boydi*i); 25.7±5.9 (S. *typhii*) |  | 1.36 mg/g crude | 0.14% |
|  |  |  |  |  | MIC- 16 mg/ml | S. *boydii*; S. *typhii* |  |  |  |
| 6 | *Vernonia amygdalina* | Leaf | Dichloromethane | Epivernodalol | Cytotoxic activity against Skin melanoma cells | 7.33 µg/ml | Owoeye et al., 2010 | 0.464 mg/g crude | 0.05% |
| 7 | *Vernonia gigantea* | Root | Ethanol | Zaluzanin C | Anti-fungal activity | 25 g/ml (*Trichophyton mentagrophytes*) | Rojas, 2000 | Not reported | Not reported |
| 8 | *Vernonia arborea* | Leaf | Hexane | Zaluzanin D;  Sesquiterpene Lactone | Anti-fungal activity | 200 ppm | Krishna Kumari et al., 2003 | 0.04% | 0.04% |
|  |  |  |  |  |  | *Rhizoctonia solani* - 100% |  |  |  |
|  |  |  |  |  |  | *Curvularia lunata* & *Botrytis* *cinerea* - 75% |  |  |  |
|  |  |  |  |  |  | *Colletotrichum lindemuthianum*, *Fusarium equisetti* & *F. Oxysporum* - 60% |  |  |  |
| 9 | *Vernonia arborea* | Leaf | Petroleum ether | Zaluzanin C | Anti-cancer activity against breast cancer cell line | Not active | Valkute et al., 2018 | - | - |
|  |  |  |  | Zaluzanin D |  | 53.7 µM - MCF7; 34.17 µM - MDA-MB-231 cell lines |  |  |  |
| 10 | *Vernonia colorata* | Leaf | Chloroform-methanol | Glycosides | Anti-inflammatory activity | 200 mg/kg (80% inhibition) | Cioffi et al., 2004 | Not reported | Not reported |
|  |  |  |  |  | Carrageenan-induced paw edema in rats |  |  |  |  |
| 11 | *Vernonia nigritiana* | Leaf | Petroleum ether | Glycosides | Topical anti-inflammatory activity | 300 µg/cm2 | Vassallo et al., 2013 | Not reported | Not reported |
|  |  |  | Chloroform | Stigmasterols |  | Not active |  |  |  |
|  |  |  | Chloroform-methanol |  |  | 300 µg/cm2 |  |  |  |

**Table S3 Anti-microbial activity of Vernonia sp. extracts against Gram positive and Gram negative bacteria**

|  | **Gram Positive** | | | | | | | | | | |  | **Gram Negative** | | | | | | | | |  |
| --- | --- | --- | --- | --- | --- | --- | --- | --- | --- | --- | --- | --- | --- | --- | --- | --- | --- | --- | --- | --- | --- | --- |
| **S. No.** | ***Vernonia* sp.** | **Part** | **Crude extract/ compound (Concentration)** | **Method** | ***S. aureus*** | ***MRSA*** | ***C. ulcerans*** | ***S. pyogenes*** | ***S. agalactiae*** | ***S. faecalis*** | ***B. subtilis*** |  | ***E. coli*** | ***S. marcescens*** | ***S. boydii*** | ***S. typhi*** | ***S. dysentriae*** | ***K. pneumoniae*** | ***P. aeruginosa*** | ***P. vulgaris*** | ***P. mirablis*** | **Reference** |
| 1 | *Vernonia cinerea* | Leaf | Hexane | AD | 10±0.76 mm |  |  |  |  |  | Nil |  | 11±0.57 mm |  |  |  |  | 11.8±1.83 mm | 9.6±0.44 mm | 10.5±0.50 mm |  | Sonibare *et al*., 2016 |
|  |  |  | Chloroform | AD | 10±0.28 mm |  |  |  |  |  | 12.6±1.36 mm |  | 12±1.36 mm |  |  |  |  | 11.7±0.72 mm | 12.8±0.72 mm | 12±0.28 mm |  |  |
|  |  |  | Ethyl acetate | AD | 10.2±0.44 mm |  |  |  |  |  | 13±0.76 mm |  | 13.5±2.02 mm |  |  |  |  | 12.8±0.44 mm | 12.7±0.72 mm | 12.8±1.09 mm |  |  |
|  |  |  |  |  |  |  |  |  |  |  |  |  |  |  |  |  |  |  |  |  |  |  |
|  |  |  | Hexane | MIC | 1.56 mg/ml |  |  |  |  |  | Nil |  | 6.25 mg/ml |  |  |  |  | 3.13 mg/ml | 3.13 mg/ml | 3.13 mg/ml |  |  |
|  |  |  |  | MBC | 3.13 mg/ml |  |  |  |  |  | Nil |  | 12.5 mg/ml |  |  |  |  | 6.25 mg/ml | 6.25 mg/ml | 6.25 mg/ml |  |  |
|  |  |  | Chloroform | MIC | 3.13 mg/ml |  |  |  |  |  | 6.25 mg/ml |  | 3.13 mg/ml |  |  |  |  | 3.13 mg/ml | 3.13 mg/ml | 1.56 mg/ml |  |  |
|  |  |  |  | MBC | 6.25 mg/ml |  |  |  |  |  | 12.5 mg/ml |  | 6.25 mg/ml |  |  |  |  | 6.25 mg/ml | 6.25 mg/ml | 3.13 mg/ml |  |  |
|  |  |  | Ethyl acetate | MIC | 6.25 mg/ml |  |  |  |  |  | 3.13 mg/ml |  | 3.13 mg/ml |  |  |  |  | 1.56 mg/ml | 6.25 mg/ml | 1.56 mg/ml |  |  |
|  |  |  |  | MBC | 12.5 mg/ml |  |  |  |  |  | 6.25 mg/ml |  | 6.25 mg/ml |  |  |  |  | 3.13 mg/ml | 12.5 mg/ml | 3.13 mg/ml |  |  |
|  |  |  |  |  |  |  |  |  |  |  |  |  |  |  |  |  |  |  |  |  |  |  |
| 2 | *Vernonia amygdalina* | Stem | Water (250 mg/ml) | AD | 6 mm |  |  |  |  |  |  |  | 6 mm |  |  |  |  |  |  |  |  | Rajasekaran et al., 2017 |
|  |  |  | Chloroform (250 mg/ml) | AD | 6 mm |  |  |  |  |  |  |  | 6 mm |  |  |  |  |  |  |  |  |  |
|  |  |  | Ethanol (250 mg/ml) | AD | 6 mm |  |  |  |  |  |  |  | 6 mm |  |  |  |  |  |  |  |  |  |
| 3 | *Vernonia amygdalina* | Flower | Chloroform | AD | 21 mm |  |  |  |  |  | 6 mm |  | 6 mm |  |  |  |  | 6 mm |  |  | 6 mm | Habtamu and Melaku, 2018 |
|  |  |  | Acetone | AD | 17 mm |  |  |  |  |  | 12 mm |  | 10 mm |  |  |  |  | 12 mm |  |  | 11 mm |  |
| 4 | *Vernonia amygdalina* | Leaf | Ethanol | AD | 11.4 mm |  |  |  |  |  |  |  | 11.3 mm |  |  |  |  | 11.7 mm | 10.8 mm |  |  | Ghamba *et al.*, 2014 |
|  |  |  | Water | AD | 11.4 mm |  |  |  |  |  |  |  | 12.5 mm |  |  |  |  | 11.8 mm | 12.2 mm |  |  |  |
|  |  |  |  |  |  |  |  |  |  |  |  |  |  |  |  |  |  |  |  |  |  |  |
|  |  |  | Ethanol | MIC | 25 mg/ml |  |  |  |  |  |  |  | 50 mg/ml |  |  |  |  | 50 mg/ml | 12.5 mg/ml |  |  |  |
|  |  |  | Water | MIC | 12.5 mg/ml |  |  |  |  |  |  |  | 50 mg/ml |  |  |  |  | 50 mg/ml | 25.0 mg/ml |  |  |  |
|  |  |  |  |  |  |  |  |  |  |  |  |  |  |  |  |  |  |  |  |  |  |  |
| 5 | *Vernonia amygdalina* | Leaf | Water | AD | 4.67±0.33 mm |  |  |  | 4.24±0.14 |  |  |  | 0 |  |  | 0 |  |  |  |  |  | Habtom and Gebrehewot, 2019 |
|  |  |  | Ethanol | AD | 17.43±0.23 mm |  |  |  | 16.3±0.23 |  |  |  | 5.76±0.15 |  |  | 6.17±0.61 |  |  |  |  |  |  |
|  |  |  | Methanol | AD | 21.50±0.29 mm |  |  |  | 19.78±0.17 |  |  |  | 4.0±0.00 |  |  | 9.5±0.23 |  |  |  |  |  |  |
|  |  |  |  |  |  |  |  |  |  |  |  |  |  |  |  |  |  |  |  |  |  |  |
|  |  |  | Water | MIC | 100 mg/ml |  |  |  | 100 mg/ml |  |  |  | Nil |  |  | Nil |  |  |  |  |  |  |
|  |  |  | Ethanol | MIC | 25 mg/ml |  |  |  | 50 mg/ml |  |  |  | 100 mg/ml |  |  | 100 mg/ml |  |  |  |  |  |  |
|  |  |  | Methanol | MIC | 50 mg/ml |  |  |  | 100 mg/ml |  |  |  | 100 mg/ml |  |  | 100 mg/ml |  |  |  |  |  |  |
| 6 | *Vernonia schimperi* | Leaf | Water (250 mg/ml) | AD | 21 mm |  |  |  |  |  |  |  | 6 mm |  |  |  |  |  |  |  |  | Rajasekaran et al., 2017 |
|  |  |  | Chloroform (250 mg/ml) | AD | 25 mm |  |  |  |  |  |  |  | 38 mm |  |  |  |  |  |  |  |  |  |
|  |  |  | Ethanol (250 mg/ml) | AD | 25 mm |  |  |  |  |  |  |  | 20 mm |  |  |  |  |  |  |  |  |  |
|  |  |  |  |  |  |  |  |  |  |  |  |  |  |  |  |  |  |  |  |  |  |  |
|  | *Vernonia schimperi* | Root | Water (250 mg/ml) | AD | 6 mm |  |  |  |  |  |  |  | 9 mm |  |  |  |  |  |  |  |  |  |
|  |  |  | Chloroform (250 mg/ml) | AD | 6 mm |  |  |  |  |  |  |  | 18 mm |  |  |  |  |  |  |  |  |  |
|  |  |  | Ethanol (250 mg/ml) | AD | 6 mm |  |  |  |  |  |  |  | 15 mm |  |  |  |  |  |  |  |  |  |
|  |  |  |  |  |  |  |  |  |  |  |  |  |  |  |  |  |  |  |  |  |  |  |
|  | *Vernonia schimperi* | Stem | Water (250 mg/ml) | AD | 6 mm |  |  |  |  |  |  |  | 6 mm |  |  |  |  |  |  |  |  |  |
|  |  |  | Chloroform (250 mg/ml) | AD | 15 mm |  |  |  |  |  |  |  | 35 mm |  |  |  |  |  |  |  |  |  |
|  |  |  | Ethanol (250 mg/ml) | AD | 13 mm |  |  |  |  |  |  |  | 22 mm |  |  |  |  |  |  |  |  |  |
|  |  |  |  |  |  |  |  |  |  |  |  |  |  |  |  |  |  |  |  |  |  |  |
| 7 | *Vernonia adoensis* | Leaf | Ethanol (25 mg/ml) | MIC | 0.21 mg/ml |  |  |  |  |  |  |  |  |  |  |  |  |  | 0.42 mg/ml |  |  | Mabhiza *et al*., 2016 |
|  |  |  |  | MBC | Nil |  |  |  |  |  |  |  |  |  |  |  |  |  | Nil |  |  |  |
|  |  |  |  |  |  |  |  |  |  |  |  |  |  |  |  |  |  |  |  |  |  |  |
| 8 | *Vernonia galamensis* | Leaf | Acetone (300 mg/ml) | AD | 24±0.6 |  |  |  |  |  |  |  | 16±0.6 |  | 27.7±0.6 | 22±1.0 |  |  |  |  |  | Tafesse et al., 2018 |
|  |  |  |  |  |  |  |  |  |  |  |  |  |  |  |  |  |  |  |  |  |  |  |
| 9 | *Vernonia tenoreana* | Leaf | Methanol | AD | 11±0.1 mm |  |  |  |  | 13.3±0.2 | 11.6±1.5 |  | 12.3±0.6 | 11.3±0.6 |  |  |  | 16.3±1.5 | 18.3±0.6 | 6.0±0.00 |  | Ogundare *et al*., 2006 |
|  |  |  | Ethylacetate | AD | 4.7±0.6 |  |  |  |  | 7.3±0.6 | 6.3±0.6 |  | 6.7±0.6 | 4.0±1.0 |  |  |  | 2.7±0.6 | 4.7±0.6 | 3.3±0.6 |  |  |
|  |  |  | Chloroform | AD | 4.7±1.2 |  |  |  |  | 4.3±0.6 | 4.6±0.6 |  | 2.3±0.6 | 4.3±0.6 |  |  |  | 2.7±0.6 | Nil | 3.7±0.6 |  |  |
|  |  |  | Hexane | AD | 3.0±0.00 |  |  |  |  | 3.0±01 | 2.7±0.6 |  | 2.0±0.00 | Nil |  |  |  | 2.3±0.6 | Nil | Nil |  |  |
|  |  |  |  |  |  |  |  |  |  |  |  |  |  |  |  |  |  |  |  |  |  |  |
|  |  | Bark | Methanol | AD | 15.7±0.6 |  |  |  |  | 7.7±2.1 | 11.7±0.6 |  | 6.3±0.6 | Nil |  |  |  | 13.3±0.6 | 13.3±0.6 | 8.3±0.6 |  |  |
|  |  |  | Ethylacetate | AD | 7.7±0.6 |  |  |  |  | 5.7±0.6 | 4.7±0.6 |  | Nil | 0 |  |  |  | 3.3±0.6 | 3.3±1.5 | 3.3±0.6 |  |  |
|  |  |  | Chloroform | AD | Nil |  |  |  |  | 4.3±1.5 | Nil |  | 2.3±0.6 | Nil |  |  |  | Nil | 2.0±1.2 | Nil |  |  |
|  |  |  | Hexane | AD | 2.0±0.00 |  |  |  |  | 3.3±0.6 | Nil |  | 2.0±0.00 | Nil |  |  |  | 2.3±0.6 | 6.0±0.00 | Nil |  |  |
|  |  |  |  |  |  |  |  |  |  |  |  |  |  |  |  |  |  |  |  |  |  |  |
|  |  | Leaf | Methanol | MIC | 10 mg/ml |  |  |  |  | 10 mg/ml | 10 mg/ml |  | 10 mg/ml | 15 mg/ml |  |  |  | 10 mg/ml | 10 mg/ml | 25 mg/ml |  |  |
|  |  |  | Ethylacetate | MIC | 10 mg/ml |  |  |  |  | 10 mg/ml | 10 mg/ml |  | 15 mg/ml | 10 mg/ml |  |  |  | 15 mg/ml | 15 mg/ml | 15 mg/ml |  |  |
|  |  |  | Chloroform | MIC | 10 mg/ml |  |  |  |  | 10 mg/ml | 15 mg/ml |  | 10 mg/ml | 15 mg/ml |  |  |  | 15 mg/ml | Nil | 15 mg/ml |  |  |
|  |  |  | Hexane | MIC | 15 mg/ml |  |  |  |  | 15 mg/ml | 15 mg/ml |  | Nil | Nil |  |  |  | 15 mg/ml | Nil | Nil |  |  |
|  |  |  |  |  |  |  |  |  |  |  |  |  |  |  |  |  |  |  |  |  |  |  |
|  |  | Bark | Methanol | MIC | 10 mg/ml |  |  |  |  | 15 mg/ml | 10 mg/ml |  | 20 mg/ml | Nil |  |  |  | 10 mg/ml | 20 mg/ml | 20 mg/ml |  |  |
|  |  |  | Ethylacetate | MIC | 10 mg/ml |  |  |  |  | 10 mg/ml | 15 mg/ml |  | Nil | Nil |  |  |  | 15 mg/ml | 15 mg/ml | 15 mg/ml |  |  |
|  |  |  | Chloroform | MIC | Nil |  |  |  |  | 15 mg/ml | Nil |  | 15 mg/ml | Nil |  |  |  | Nil | 15 mg/ml | Nil |  |  |
|  |  |  | Hexane | MIC | 15 mg/ml |  |  |  |  | 15 mg/ml | Nil |  | 15 mg/ml | Nil |  |  |  | 15 mg/ml | 15 mg/ml | Nil |  |  |
|  |  |  |  |  |  |  |  |  |  |  |  |  |  |  |  |  |  |  |  |  |  |  |
| 10 | *Vernonia colorata* | Leaf | Hexane | AD | 13.00 mm |  |  |  |  |  |  |  |  |  |  |  |  |  | Nil |  |  | Julien *et al*., 2012 |
|  |  |  |  | MIC | 3.12 mg/ml |  |  |  |  |  |  |  |  |  |  |  |  |  | 25.00 mg/ml |  |  |  |
|  |  |  |  |  |  |  |  |  |  |  |  |  |  |  |  |  |  |  |  |  |  |  |
| 11 | *Vernonia ambigua* | Whole plant | Ethanol (100 mg/ml) | DD | 18 mm | 14 mm | 16 mm | 16 mm |  |  |  |  |  |  |  | 20 mm | Nil | 16 mm | 18 mm |  | Nil | Aliyu *et al*., 2011 |
|  |  |  |  | MIC | 2.5 mg/ml | 2.5 mg/ml | 2.5 mg/ml | 2.5 mg/ml |  |  |  |  |  |  |  | 1.25 mg/ml | Nil | Nil | 1.25 mg/ml |  | Nil |  |
|  |  |  |  |  |  |  |  |  |  |  |  |  |  |  |  |  |  |  |  |  |  |  |
|  |  |  | Chloroform (100 mg/ml) | DD | 20 mm | 20 mm | 19 mm | 18 mm |  |  |  |  |  |  |  | 22 mm | Nil | 19 mm | 20 mm |  | Nil |  |
|  |  |  |  | MIC | 1.25 mg/ml | 1.25 mg/ml | 2.5 mg/ml | 2.5 mg/ml |  |  |  |  |  |  |  | 1.25 mg/ml | Nil | Nil | 1.25 mg/ml |  | Nil |  |
|  |  |  |  |  |  |  |  |  |  |  |  |  |  |  |  |  |  |  |  |  |  |  |
| 12 | *Vernonia blumeoides* | Whole plant | Ethanol (100 mg/ml) | DD | 22 mm | 17 mm | 24 mm | 16 mm |  |  |  |  |  |  |  | 18 mm | 14 mm | 22 mm | Nil |  | Nil | Aliyu et al., 2011 |
|  |  |  |  | MIC | 1.25 mg/ml | 1.25 mg/ml | 1.25 mg/ml | 2.5 mg/ml |  |  |  |  |  |  |  | 2.5 mg/ml | 2.5 mg/ml | 2.5 mg/ml | Nil |  | Nil |  |
|  |  |  |  |  |  |  |  |  |  |  |  |  |  |  |  |  |  |  |  |  |  |  |
|  |  |  | Chloroform (100 mg/ml) | DD | 24 mm | 20 mm | 27 mm | 18 mm |  |  |  |  |  |  |  | 20 mm | 18 mm | 27 mm | Nil |  | Nil |  |
|  |  |  |  | MIC | 1.25 mg/ml | 1.25 mg/ml | 1.25 mg/ml | 2.5 mg/ml |  |  |  |  |  |  |  | 1.25 mg/ml | 2.5 mg/ml | 2.5 mg/ml | Nil |  | Nil |  |
|  |  |  |  |  |  |  |  |  |  |  |  |  |  |  |  |  |  |  |  |  |  |  |
| 13 | *Vernonia Oocephala* | Whole plant | Ethanol (100 mg/ml) | DD | 18 mm | 20 mm | 20 mm | 16 mm |  |  |  |  |  |  |  | Nil | Nil | 20 mm | Nil |  | 22 mm | Aliyu *et al*., 2011 |
|  |  |  |  | MIC | 2.5 mg/ml | 1.25 mg/ml | 1.25 mg/ml | 2.5 mg/ml |  |  |  |  |  |  |  | Nil | Nil | Nil | Nil |  | 1.25 mg/ml |  |
|  |  |  |  |  |  |  |  |  |  |  |  |  |  |  |  |  |  |  |  |  |  |  |
|  |  |  | Chloroform (100 mg/ml) | DD | 20 mm | 20 mm | 22 mm | 24 mm |  |  |  |  |  |  |  | Nil | Nil | 25 mm | Nil |  | 27 mm |  |
|  |  |  |  | MIC | 1.25 mg/ml | 1.25 mg/ml | 1.25 mg/ml | 1.25 mg/ml |  |  |  |  |  |  |  | Nil | Nil | NIl | Nil |  | 1.25 mg/ml |  |
|  |  |  |  |  |  |  |  |  |  |  |  |  |  |  |  |  |  |  |  |  |  |  |
| 14 | *Vernonia cinerea* | Leaf | Petroleum ether (100 mg/ml) | DD | 12.46±0.058 |  |  |  |  |  | Nil |  | 11.033±0.058 |  |  | Nil |  |  | Nil |  |  | Somasundaram, 2010 |
|  |  |  |  |  |  |  |  |  |  |  |  |  |  |  |  |  |  |  |  |  |  |  |
|  |  |  | Ethanol (100 mg/ml) | DD | 13.40±0.100 |  |  |  |  |  | 14.033±0.058 |  | 14.483±0.029 |  |  | 13.50±0.00 |  |  | 12.417±0.076 |  |  |  |
|  |  |  |  |  |  |  |  |  |  |  |  |  |  |  |  |  |  |  |  |  |  |  |
|  |  |  | Water (100 mg/ml) | DD | 15.033±0.058 |  |  |  |  |  | ; |  | 21.033±0.058 |  |  | 16.033±0.058 |  |  | 19.100±0.10 |  |  |  |

Method: DD- Disc Diffusion assay; ADA- Agar Diffusion Assay; MIC- Minimum Inhibitory Concentration; MBC- Minimum Bactericidal Concentration

**Table S4 Quantification of phytoconstituents in the hexane leaf extracts of *V. arborea.***

| **Content in leaf extract** | **Expression Unit** | **Results** |
| --- | --- | --- |
| Total phenol content | mg Gallic acid equivalent/g extract | 752.5 |
| Total steroid content | mg β-sitosterol equivalent/g extract | 205.75 |
| Total terpenoid content | mg Linalool equivalent/g extract | 61.56 |
| Total tannin content | mg Tannic acid equivalent/g extract | 41.83 |

**Table S5 Wound closure of infected tissues observed in adult zebrafish treated with 0.5% V. *arborea* fractions and control groups.** Wound closure (WC) was 3-fold better in treated groups compared to untreated ones. Values were significant with p<0.05.

| **Observation time** | **Wound diameter [mm] [mean ±SD]** | | | | | | |
| --- | --- | --- | --- | --- | --- | --- | --- |
|  | **Test fractions [0.5% W/W]** | | | | **Positive control** | **Untreated control** | **Vehicle control** |
|  | **F10** | **F26** | **F28** | **F30** | **[PC]** | **[UC]** | **[VC]** |
| **0 hrs** | 3.02±0.10 | 3.00±0.17 | 2.95±0.05 | 3.06±0.03 | 3.06±0.02 | 3.02±0.04 | 3.03±0.03 |
| **5 dpw** | 2.25±0.13 | 2.46±0.12 | 2.80±0.05 | 2.90±0.04 | 2.15±0.05 | 3.06±0.08 | 2.80±0.05 |
| **10 dpw** | 2.03±0.10 | 2.13±0.10 | 2.65±0.10 | 2.70±0.04 | 2.03±0.10 | 3.00±0.05 | 2.75±0.05 |
| **15 dpw** | 1.45±0.05 | 2.05±0.06 | 2.30±0.03 | 1.95±0.12 | 1.80±0.04 | 2.35±0.06 | 2.30±0.05 |
| **20 dpw** | 1.10±0.13 | 2.05±0.06 | 2.00±0.07 | 1.30±0.04 | 1.25±0.04 | 2.15±0.10 | 1.80±0.05 |
| **25 dpw** | 0.30±0.04 | 1.70±0.03 | 1.75±0.05 | 0.85±0.06 | 0.45±0.10 | 2.05±0.03 | 1.80±0.06 |
| **WC % 25 dpw** | 92 | 44 | 41 | 72 | 84 | 32 | 41 |

**References pertaining to supplementary data:**

1. Iliyasu MY, Yakubu MN, Ladan MA, Sahal MR, Joshua RK. Evaluation of phytochemical and in vitro anti-dermotophyte activity of *Vernonia amygdalina* (bitter leaf) locally used in the treatment of ringworm infection. Int J Med Clin Imag. 2019;5(1):73-79. <https://www.researchgate.net/publication/345918209>.
2. Iyanuloluwa O, Adamu KY & Audu JA. Antibacterial and antifungal activities of aqueous leaves extract of some medicinal plants. GSC Biol Pharmaceut Sci. 2019;9(1):62-69. <https://doi.org/10.30574/gscbps.2019.9.1.0185>.
3. Da Cruz JER, da Costa Guerra JF, de Souza Gomes M, Freitas GRO, Morais ER. Phytochemical Analysis and Evaluation of Antimicrobial Activity of *Peumus boldus, Psidium guajava, Vernonia polysphaera, Persea Americana, Eucalyptus citriodora* Leaf Extracts and *Jatropha multifida* Raw Sap. Curr Pharmaceut Biotechnol. 2019;20(5):433-444. <https://doi.org/10.2174/1389201020666190409104910>.
4. Thomas E, Gopalakrishnan V, Somasagara RR, Choudhary B, Raghavan SC. Extract of *Vernonia condensata*, inhibits tumor progression and improves survival of tumor-allograft bearing mouse. Scien Rep. 2016;6:23255. <https://doi.org/10.1038/srep23255>.
5. Khalafalla MM, Abdellatef E, Daffalla HM, Nassrallah AA, Aboul-Enein KM, Lightfoot DA, et al. Antileukemia activity from root cultures of *Vernonia amygdalina.* J Med Plant Res. 2009;3(8):556-562. <http://www.academicjournals.org/JMPR>.
6. Choudary S, Sharma M, Tripati J, Mishra P. Antihyperglycemic activity of *Vernonia cinerea* L. on alloxan-induced diabetic mice. Int J Adv Res. 2013;1(2):35-42. <http://www.journalijar.com/uploads/2013-04-23_150310_533.pdf>.
7. Alara OR & Abdurahman NH Anti-diabetic activity and mineral elements evaluation of *Vernonia amygdalina* leaves obtained from Malaysia. J Res Pharm. 2019;23(3):514-521. <https://doi.org/10.12991/jrp.2019.158>.
8. Manjunatha, BK, Vidya SM, Rashmi KV, Mankani KL, Shilpa HJ, Jagadeesh Singh SD. Evaluation of wound-healing potency of *Vernonia arborea* Hk. Ind J Pharmacol. 2005;37(4):223-226. <http://www.bioline.org.br/pdf?ph05057>.
9. Vaidyanathan, L & Lokeswari TS. Compounds from *Vernonia arborea* Buch.-Ham. Inhibit microbes that impair wound healing. J Pharm Res Int. 2021;33(44B):103-113. <https://doi.org/10.9734/jpri/2021/v33i44B32655>.
10. Vaidyanathan L & Sivaswamy LT. Cutaneous wound healing by *Vernonia arborea* extracts in adult zebrafish model. Int J Pharm Sci Res. 2022;13(12):4952-62.
11. Leite SN, Palhano G, Almeida S & Biavatti MW. Wound healing activity and systemic effects of *Vernonia scorpioides* extract in guinea pig. Fitoter. 2002;73(6):496-500. <https://doi.org/10.1016/s0367-326x(02)00169-7>.
12. Nafiu AB, Akinwale OC, Akinfe OA, Owoyele BV, Abioye AIR, Abdulazeez FI, et al. Histomorphological evaluation of wound healing - comparison between use of honey and *Vernonia amygdalina* leaf juice. Trop J Health Sci. 2016;23(3):32-38. https://www.ajol.info/index.php/tjhc/article/view/149877.
13. Kupchan SM, Hemingway RJ, Karim A, Werner D. Tumor inhibitors. XLVII. Vernodalin and vernomygdin, two new cytotoxic sesquiterpene lactones from *Vernonia amygdalina* Del. J Org Chem. 1969;34:3908‑11. <https://pubs.acs.org/doi/10.1021/jo01264a035>.
14. Abhay SM, Lucantoni L, Dahiya N, Dori G, Dembo EG, Esposito F, et al. Plasmodium transmission blocking activities of *Vernonia amygdalina* extracts and isolated compounds. Malaria J. 2015;25:14, 288. <https://pubmed.ncbi.nlm.nih.gov/26208861>.
15. Tafesse G, Mekonnen Y, Makonnen E, Majinda RRT, Bojase-Moleta G, Yeboah SO. Antibacterial activity of crude extracts and pure compounds isolated from *Vernonia galamensis* leaves. Afr J Pharm Pharmacol. 2018;12(11):136-141.
16. Owoeye O, Yousuf S, Nadeem Akhtar M, Qamar K, Dar A, Farombi EO, et al. Another anticancer elemanolide from *Vernonia amygdalina* Del. Int J Biol Chem Sci. 2010;4(1):226-234. <http://ajol.info/index.php/ijbcs>.
17. Rojas R. Secondary metabolites of *Vernonia gigantea* roots: isolation, antimicrobial, chemical and biotransformation studies. Dissertation The Ohio State University. 2000.
18. Krishna Kumari GN, Masilamani S, Ganesh MR, Aravind S, & Sridhar SR. Zaluzanin D: a fungistatic sesquiterpene from *Vernonia arborea*. Fitoterapia. 2003;74:479-482. <https://pubmed.ncbi.nlm.nih.gov/12837366>.
19. Valkute TR, Aratikatla EK, Gupta NA, Ganga S, Santra MK, Bhattacharya AK. Synthesis and anticancer studies of Michael adducts and Heck arylation products of sesquiterpene lactones, zaluzanin D and zaluzanin C from *Vernonia arborea*. RSC Adv. 2018;8(67):38289-38304. <https://pubmed.ncbi.nlm.nih.gov/35559>
20. Cioffi G, Sanogo R, Diallo S, Romussi G, Tommasi ND. New compounds from an extract of *Vernonia colorata* leaves with anti-inflammatory activity. J Nat Prod. 2004;67(3):389-94. <https://pubmed.ncbi.nlm.nih.gov/15043416>.
21. Vassallo A, Tommasi ND, Merfort I, Sanogo R, Severino L, Pelin M, et al. Steroids with anti-inflammatory activity from *Vernonia nigritiana* Oliv. & Hiern. Phytochem. 2013;96:288-298. https://doi.org/10.1016/j.phytochem.2013.09.002.
22. Sonibare MA, Aremu OT, Okorie PN. Antioxidant and antimicrobial activities of solvent fractions of *Vernonia cinerea* (L.) Less leaf extract. Afr Health Sci. 2016;16(2):629-639. <https://doi.org/10.4314%2Fahs.v16i2.34>.
23. Rajasekaran R, Asefaw Y, Gabrekidan Y, Medhanie G, Yamane B. Antimicrobial activity of *Vernonia schimperi* and *Vernonia amygdalina* against selected clinical pathogens. Int J Pharmaceut Sci Rev Res. 2017;47(1):141-144. <https://www.researchgate.net/publication/326504831>.
24. Habtamu A. & Melaku Y. Antibacterial and antioxidant compounds from the flower extracts of *Vernonia amygdalina*. Advances in pharmacological sciences. 2018;20:4083736. <https://doi.org/10.1155/2018/4083736>.
25. Mabhiza D, Chitemerere T, & Mukanganyama S. Antibacterial properties of alkaloid extracts from *Callistemon citrinus* and *Vernonia adoensis* against Staphylococcus aureus and Pseudomonas aeruginosa. Int J Med Chem. 2016;6304163. <https://doi.org/10.1155/2016/6304163>.
26. Mozirandi W. & Mukanganyama S. Antibacterial activity and mode of action of *Vernonia adoensis* (Asteraceae) extracts against *Staphylococcus aureus* and *Pseudomonas aeruginosa*. J Biolog Act Prod Nat. 2017;7(5). <https://doi.org/10.1080/22311866.2017.1378922>.
27. Ghamba PE, Balla H, Goje LJ, Halidu A, & Dauda MD. In vitro antimicrobial activities of *Vernonia amygdalina* on selected clinical isolates. Int J Curr Microbiol App Sci. 2014;3(4):1103-1113.
28. Habtom S, Gebrehiwot S. In vitro antimicrobial activities of crude extracts of *Vernonia amygdalina* and *Croton macrostachyus* against some bacterial and fungal test pathogens. J Phytopharm. 2019;8(2):57-62.
29. Ogundare A, Adetuyi F & Akinyosoye F. Antimicrobial activities of *Vernonia tenoreana*. Afr J Biotech. 2006;85646373. <https://doi.org/10.4314/AJB.V5I18.55815>.
30. Julien KG & Siaka S. Phytochemical assessment and antimicrobial activity of leaves extract of *Vernonia colorata* (Wild.) Drake on resistant germs of *Staphylococcus aureus* and *Pseudomonas aeruginosa*. J Chem Pharm Res. 2012;4(5):2490-2494. <https://www.researchgate.net/publication/289239511>.
31. Aliyu AB, Musa AM, Abdullahi MS, Ibrahimi H, Oyewale AO. Phytochemical screening and antibacterial activities of *Vernonia ambigua*, *Vernonia* *blumeoides* and *Vernonia oocephala* (Asteraceae). Acta Polon Pharmaceut. 2011;68(1):67-73. <https://pubmed.ncbi.nlm.nih.gov/21485703>.
32. Somasundaram A, Velmurugan V, & Senthilkumar GP. In vitro antimicrobial activity of *Vernonia cinerea* (L) Less. Pharmacol online. 2010;2:957-960. <https://pharmacologyonline.silae.it/files/newsletter/2010/vol2/102>.
